# A New *In Vitro* Model to Study Cellular Responses after Thermomechanical Damage in Monolayer Cultures

**DOI:** 10.1371/journal.pone.0082635

**Published:** 2013-12-09

**Authors:** Alice Hettler, Simon Werner, Stefan Eick, Stefan Laufer, Frank Weise

**Affiliations:** 1 Department Molecular Biology, Natural and Medical Sciences Institute at the University of Tübingen, Reutlingen, Germany; 2 Department Bio-Microelectromechanical Systems / Sensors, Natural and Medical Sciences Institute at the University of Tübingen, Reutlingen, Germany; 3 Aesculap AG, Tuttlingen, Germany; 4 Department Pharmaceutical Chemistry, Institute of Pharmaceutical Sciences, Eberhard-Karls-Universität Tübingen, Tübingen, Germany; Dalhousie University, Canada

## Abstract

Although electrosurgical instruments are widely used in surgery to cut tissue layers or to achieve hemostasis by coagulation (electrocautery), only little information is available concerning the inflammatory or immune response towards the debris generated. Given the elevated local temperatures required for successful electrocautery, the remaining debris is likely to contain a plethora of compounds entirely novel to the intracorporal setting. A very common *in vitro* method to study cell migration after mechanical damage is the scratch assay, however, there is no established model for thermomechanical damage to characterise cellular reactions. In this study, we established a new *in vitro* model to investigate exposure to high temperature in a carefully controlled cell culture system. Heatable thermostat-controlled aluminium stamps were developed to induce local damage in primary human umbilical vein endothelial cells (HUVEC). The thermomechanical damage invoked is reproducibly locally confined, therefore allowing studies, under the same experimental conditions, of cells affected to various degrees as well as of unaffected cells. We show that the unaffected cells surrounding the thermomechanical damage zone are able to migrate into the damaged area, resulting in a complete closure of the ‘wound’ within 48 h. Initial studies have shown that there are significant morphological and biological differences in endothelial cells after thermomechanical damage compared to the mechanical damage inflicted by using the unheated stamp as a control. Accordingly, after thermomechanical damage, cell death as well as cell protection programs were activated. Mononuclear cells adhered in the area adjacent to thermomechanical damage, but not to the zone of mechanical damage. Therefore, our model can help to understand the differences in wound healing during the early phase of regeneration after thermomechanical vs. mechanical damage. Furthermore, this model lends itself to study the response of other cells, thus broadening the range of thermal injuries that can be analysed.

## Introduction

Since the middle of the last century, electrosurgery has become routine in open and minimal invasive surgeries: it is used to achieve division of tissue [1], and has most recently been employed for more advanced applications such as blood vessel sealing and hemostasis [2–5]. Besides mechanical interventions such as suturing or stapling, sealing by using bipolar current offers an alternative for blood vessel closure in surgeries, resulting in a low total operative time and reduced blood loss [6,7]. Nevertheless, due to the sealing process, different inflammatory responses have been previously observed [8], and it is not well understood whether these interventions invoke a response of the patient’s immune system. The objective of this study was to establish a new *in vitro* model of thermomechanical damage which mimics cell damage caused by electrosurgery, as an alternative to *in vivo* studies on wound healing after tissue fusion. During electrocautery, the majority of tissues affected are blood vessels. The inner surface of blood vessels is lined with the endothelium, a monolayer of endothelial cells. Human umbilical vein endothelial cells (HUVEC) are widely employed for analysis of wound healing and inflammation *in vitro*, and we therefore chose these cells for our investigation. The endothelium plays an important role including the regulation of thrombosis, platelet adherence and regulation of immune and inflammatory responses [9]. A hallmark for an ongoing process of wound healing is the adherence of leukocytes, neutrophiles and monocytes to the endothelium. Endothelial cells express cell surface molecules that attract circulating blood cells to the site of injury[10–12]. The model described in this article is characterised by the highly reproducible formation of locally confined damage zones, in which the thermomechanical damage inflicted elicits a cellular resoponse that ultimately results in the adhesion of mononuclear cells without additional administration of cytokines. Therefore, this model is suitable to investigate modulators of the initial steps of wound healing after thermal damage.

## Materials and Methods

### Thermo mechanical (thermal) and mechanical damage set-up

**Figure 1:**
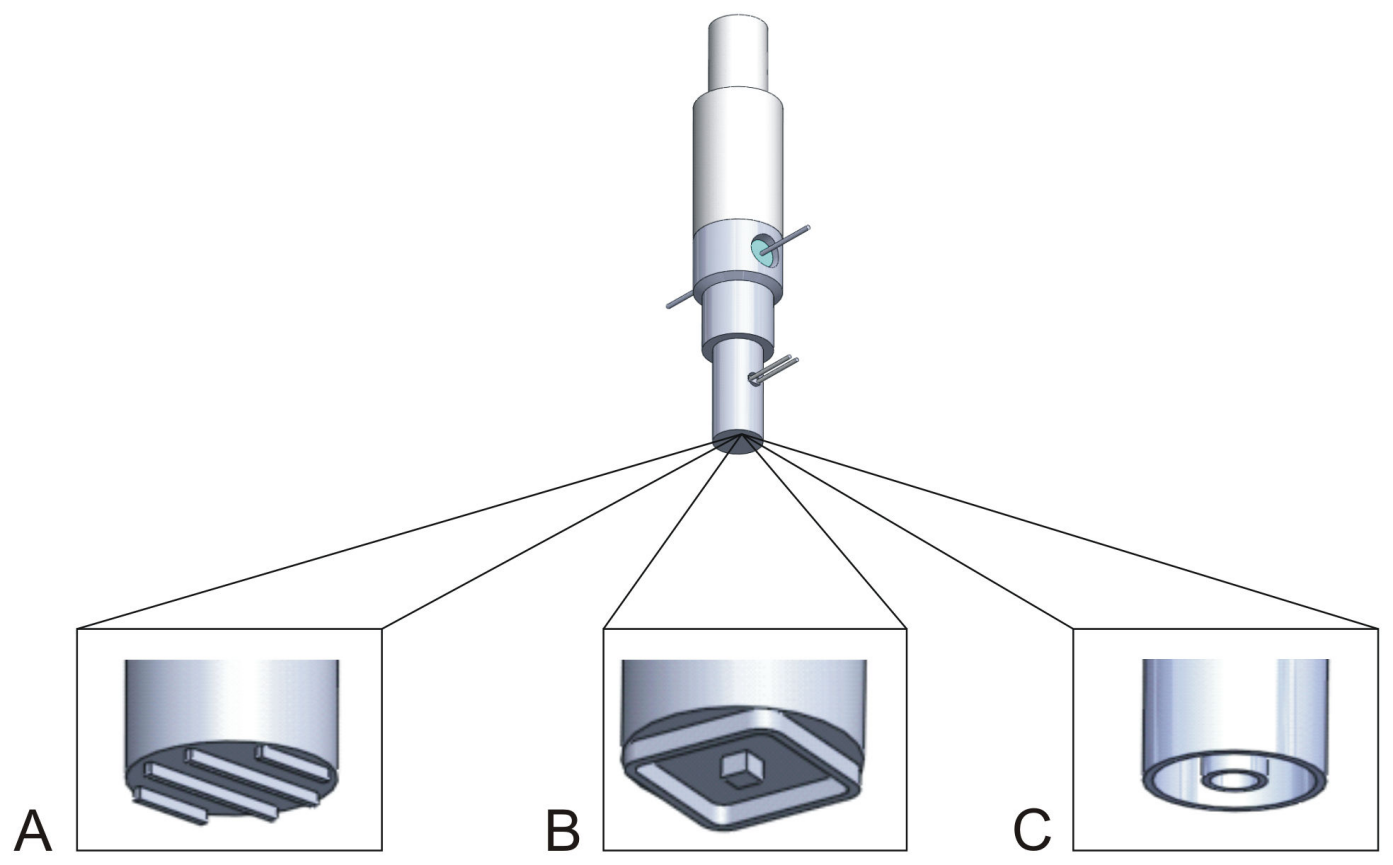
Three types of the thermo-mechanical stamp with different contour geometry. A: four-line stamp: length of outer lines: 4.5 mm and inner lines 8.5 mm. B: square stamp (length 6,5 mm) with central square (length 1 mm), C: two-ring stamp (two concentric rings): outer ring: Ø 8,5 mm inner ring Ø 3 mm. Width of all contact surfaces: approx. 400 µm; space between the contact surfaces: 2.3 mm.

**Figure 2:**
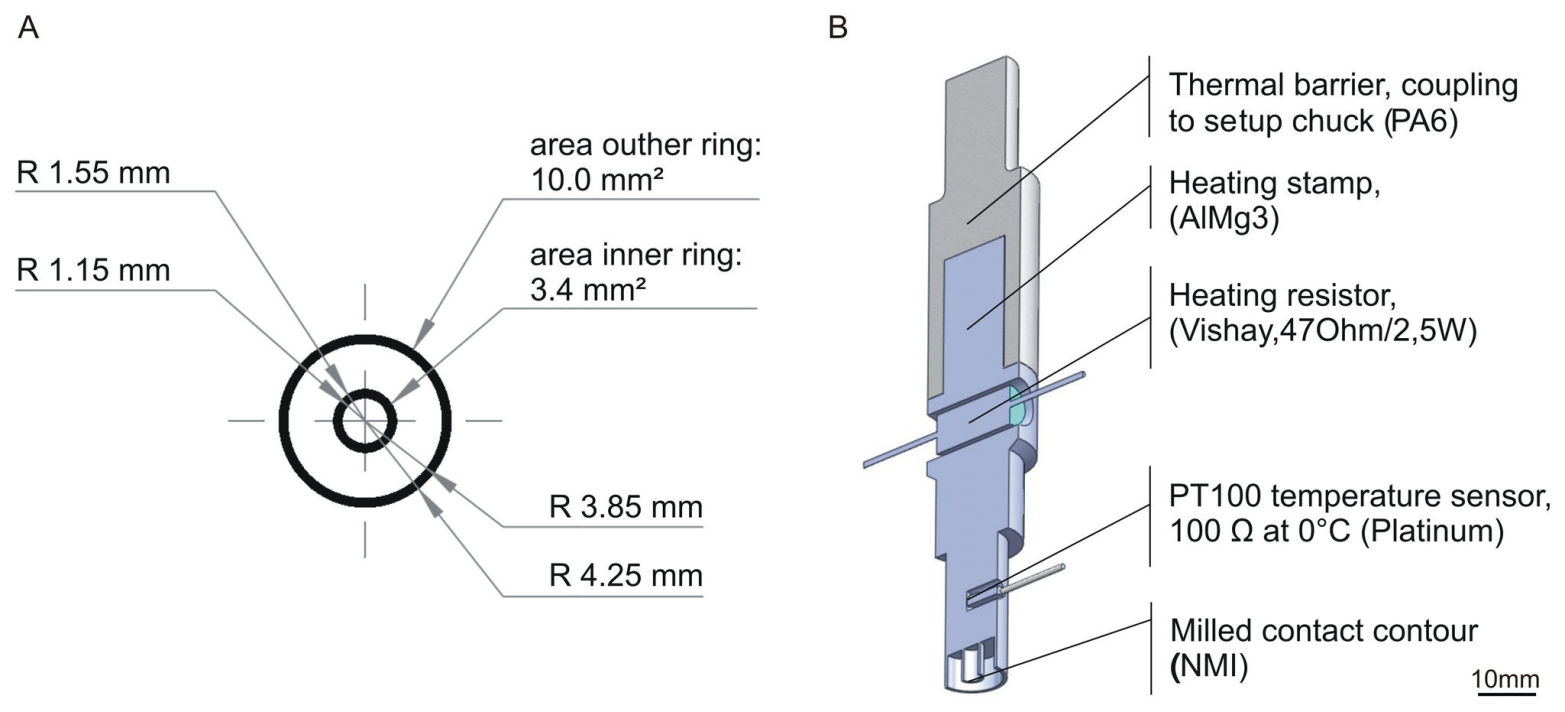
Technical data of the two-ring stamp. A: Schematic illustration with dimensions of the two rings (drawing not to scale).R = radius. B: cross section of the heating stamp with integrated thermal resistor and temperature resistor.

To induce locally-confined damage to the HUVEC cell monolayer, a heatable aluminum stamp was developed. The key aspects for the design were the contour of the contact area, a reproducible, quick recovery of the stamp temperature after contacting the cells for repeated stamping, and resistance to solvents for easy cleaning. Stable heating was achieved using a 47 Ω/ 5 W thermal resistor integrated in the shaft of the stamp. The resistor was fixed by a two component thermal polyamide glue (Electrolube, Leicestershire, UK) to ensure a fast heat-up process. In order to measure and stabilise the stamp temperature at the contact area, a PT100 resistor was used together with a 20 W TCX-1 temperature controller (Multichannel Systems, Reutlingen, Germany). This PT 100 resistor was positioned close to the front of the stamp to measure the contact area’s temperature and was similarly fixed by the thermal glue. A polyamide (Ertalon; 66-GF30,Quadrant Epp,Germany) was integrated to prevent a significant thermal flow into the set-up chuck, thus preventing unstable thermal control and simplifying the heat up process. The thermal conductivity (λ~0.3 W/m K) of this polyamide is approx. 500 times lower than that of the aluminum alloy stamp (5754-HIII; Aalco Metals Ltd. Cobham, UK, λ=147 W/m K), therefore rendering it an appropriate material for mechanical coupling to the set-up chuck. The function of this polyamide is to serve as a thermal barrier while remaining resistant to thermomechanical deformation. The melting point (260 °C) of the polyamide glue is sufficiently high to allow the instrument to be used in a wide range of temperatures, encompassing the range usually applied during electrocautery. As an experimental platform for all cultures, a commercially available 24 well plate (Corning Incorporated, NY, USA) was used with a cover slip (diameter d=12 mm) inserted into each of the wells to prevent thermomechanical damage (hereafter referred to as “thermal” damage) to the cell culture plastic material. The shape and the diameter of the thermomechanical stamp were adjusted to the diameter of the cover slip, leaving an edge of approx. 1.8 mm. To investigate the influence of the contour geometry, three alternative types of thermomechanical stamps were designed as shown in (Figure 1). The four-line contour was intended to be as comparable to the established scratching procedure [13] as possible within our setup. The square stamp was created to combine long linear structures and a small point damage source, while the two-ring stamp was selected to provide the highest damage area to surface ratio. The technical data of the two-ring stamp is summarized as an example in (Figure 2).

**Figure 3:**
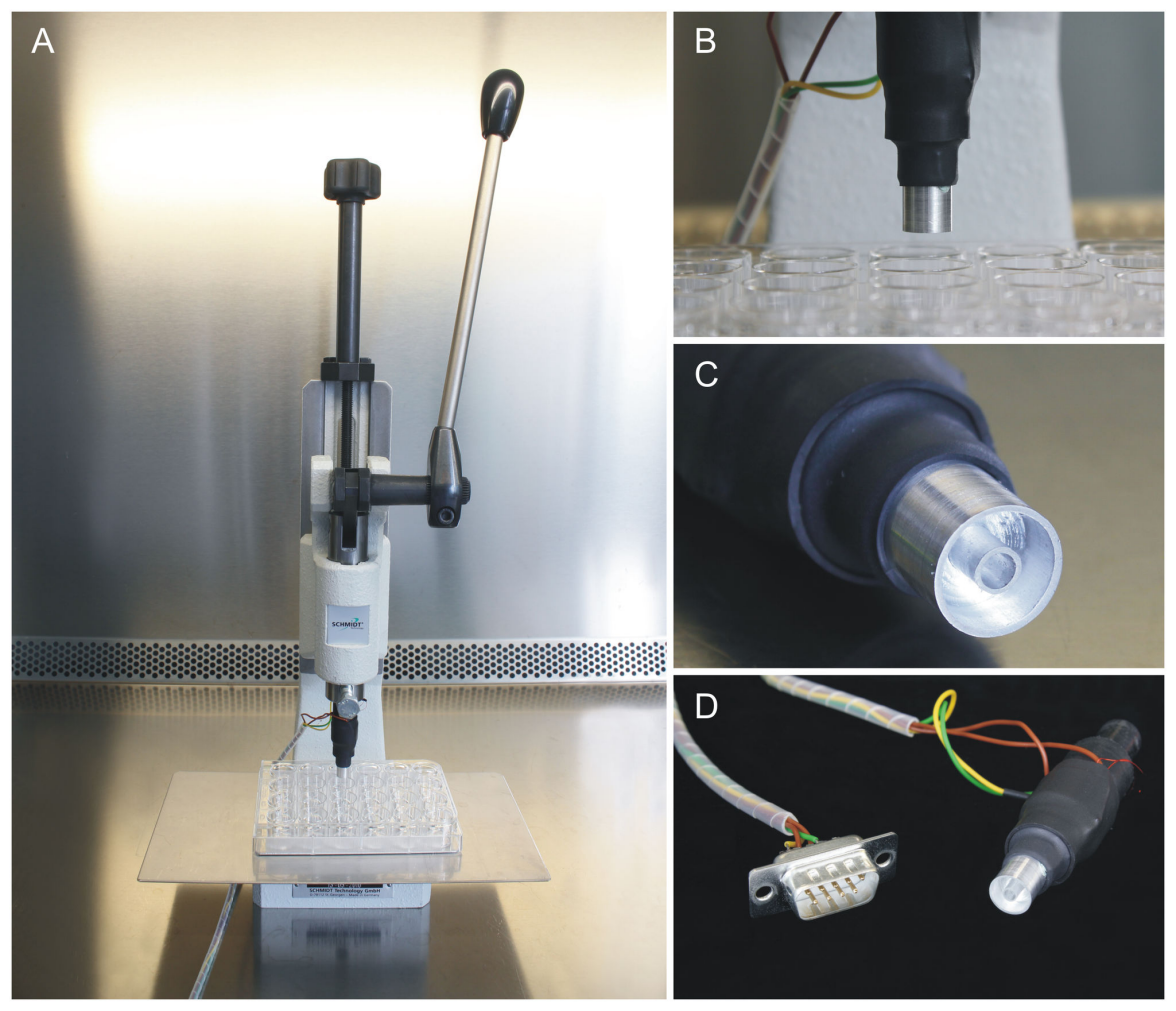
Setup for thermal damage on monolayer cultures. A: the manual press, B: the thermal mechanical stamp situated above a 24 well plate, C) detail of the final two-ring stamp contour, D: stamp and cabling.

For application, one of the alternative aluminum stamps was inserted into the chuck of a manual press (Schmidt Technology, Germany) and connected to a temperature controller and a PC. During the course of an experiment, the well plate was moved horizontally and/or vertically to sequentially stamp each of the wells. The setup is shown in (Figure 3).

### Temperature setting and stability

**Figure 4:**
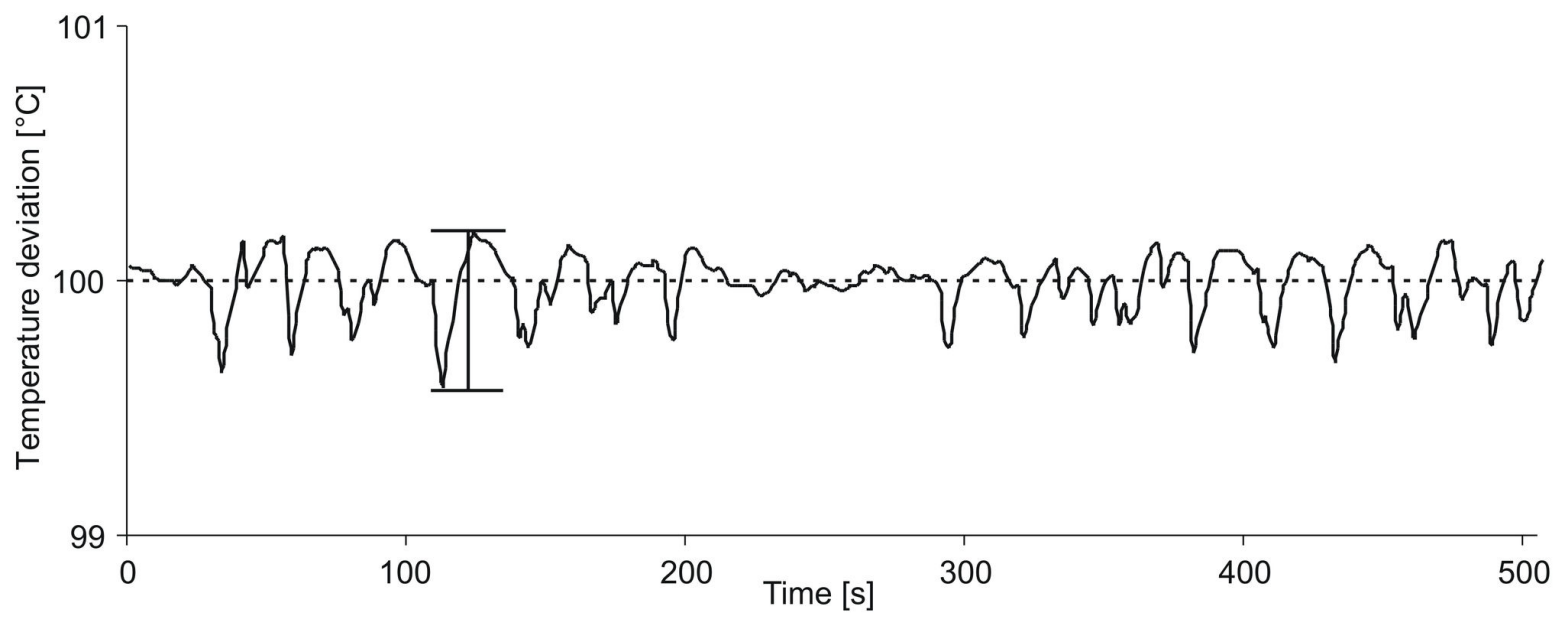
Temperature deviation during contact of the two-ring stamp with the cell culture. The spikes in the diagram show temperature deviation as an average of three measurements. The maximum deviation was +0.18 °C and -0.42 °C referred to set point temperature, the calculated standard deviation was 0.18 °C.

After connecting the cable set, the whole stamp was isulated with a shrinking tube (polyolephine) to limit thermal radiation and thermal losses due to convective flow of heat. Combined with the thermal barrier coupling, this set-up ensures the heat is preferentially guided to the non-insulated contact contour. A constant measurement with two independent PT100 sensors was performed to confirm that the temperature in the inner ring structure is comparable to the bulk temperature. The measured difference between bulk and contact temperature was found to be stable 10 minutes after starting the heat up process at ∆T_const_ = 3.5 °C. Therefore this difference treated as an offset, which had to be subtracted from the desired temperature value of the TCX-1 controller yielding the true contact area temperature. In the following, the temperature indicated always referrs to the bulk temperature. Figure 4 shows the temperature stability during an experiment versus the stamp-to-cell-culture contact time as an average of three measurements. The downward-pointing spikes are due to the cooling during contact of the two-ring stamp with the cell culture. The calculated standard deviation of 0.18 °C confirms that even after repeated stamping there is little deviation from the set temperature value.

### Cell culture

Primary human umbilical vein endothelial cells (HUVEC) were obtained from PromoCell (Heidelberg, Germany) and were cultured in VascuLife® basal medium supplemented with VascuLife® VEGF Life Factors® Kit (Lifeline Cell Technology GmbH, Troisdorf, Germany). HUVEC were cultured at 37 °C in humidified air containing 5 % CO_2_ and were passaged using the DetachKit (PromoCell, Heidelberg, Germany) according to the manufacturer’s recommendations. For the studies, only cells at passage 3-6 were used. For all experiments, HUVEC were seeded on collagen-coated 12 mm cover slips (collagen: BD Biosiences, Heidelberg, Germany; cover slips: Glaswarenfabrik Karl Hecht GmbH and Co KG, Sondelheim, Germany) in 24 well plates with hydrocortisone-free VascuLife® basal medium. Only monolayers that were tightly confluent were used for experiments. The human monocytic cell line THP-1 was originally obtained from LGC Standards (Wesel, Germany). Additionally, this cell line was retrovirally transduced to express enhanced green fluorescent protein (eGFP). The THP-1 were cultured in RPMI 1640 supplemented with 10 % FBS, l-glutamine (2 mM), β-mercaptoethanol (20 µM) (all obtained from PAA Laboratories GmbH, Pasching, Austria), 25 µg/ml hygromycin (Merck Millipore, Darmstadt, Germany) and maintained at 37 °C in humidified air containing 5 % CO_2_. Peripheral blood mononuclear cells (PBMC) from a single donor were freshly isolated by Ficoll-gradient centrifugation (specific density, 1.077 g/ml) and were used immediately after isolation for the adhesion experiments. The cells were co-cultivated for at least one hour on HUVEC in RPMI 1640 (Gibco Invitrogen ™, Life Technologies GmbH, Darmastadt, Germany) supplemented with 10 % plasma gained from the same healthy donor.

### Thermomechanical and Mechanical Damage using the different Stamping Devices

HUVEC were thermomecanically (hereafter referred to as “thermally”) affected with different stamping devices at 100 °C for 1 sec. As a control for the mechanical damage, the stamps were used at room temperature. In both cases pressure was customized manually with an average of about 3-5 bar. Before stamping, medium was removed. After stamping, the monolayer was washed once with medium equilibrated at 37 °C, and then cultured in hydrocortisone-free medium for the times indicated.

### Cell viability assay

Cell viability was determined using calcein AM (Calcein acetoxy-methyl ester; Life Technologies GmbH, Darmstadt, Germany) for live cell staining and propidium iodide (Sigma-Aldrich GmbH, Steinheim, Germany) for dead cell staining. Calcein AM is a membrane permeable marker which is virtually non-fluorescent. If the ester bond is unspecifically hydrolised by endogeneous esterases, it is transformed into green fluorescent calcein (ca. 490 nm excitation). Thus, the green fluorescence is a marker for an intact membrane and for esterase activity [14]. In contrast, propidium iodide enters only non-viable cells and intercalates with the nucleic acid. Therefore, the red fluorescence (536 nm exitation) is a marker of dead cells [14,15]. Immediately after cell damage using the different stamping devices, the medium was removed and HUVEC were incubated with 1µg/ml Calcein-AM in medium without hydrocortisone for 10 min at 37 °C in humidified air containing 5 % CO_2_. After incubation, the cells were washed twice with warm medium and were incubated with 10 µg/ml propidium iodide for one additional minute at room temperature. After the staining, cells were washed twice with medium equilibrated at 37 °C. Cell viability was determined immediately after staining (approximately 30 min after cell damage) using a microscope (Axiovert 200M, Carl Zeiss AG, Oberkochen, Germany). To avoid cell death due to long duration of microscopy, plates were analysed within 30 min. Sample collection therefore was in between 30 min and 1 h after damage. For long time experiments (up to 50 h), damaged HUVEC were washed twice with warm medium and incubated at 37 °C in humidified air containing 5 % CO_2_. After incubation, cells were stained for live cells as described above.

### Migration assay

To investigate the potential of HUVEC cells adjacent to the area affected by thermal damage caused by the two-ring stamp, migration assays were performed. For the analysis of migration, cells were stained with Calcein-AM as described above 0, 20, 30, and 50 h after damaging. The migration of the cells was analysed by fluorescence microscopy (Axiovert 200M, Carl Zeiss AG, Oberkochen, Germany). Images were taken and the closure of the affected area was determined by measuring the remaining width using the Axiovision 4.8 software (Carl Zeiss AG, Oberkochen, Germany). 

### Apoptosis and necrosis double-staining

To analyse whether cells undergo apoptosis or necrosis after thermal damage, a double immunofluorescence staining was performed using the Annexin V-EGFP Apoptosis Detection Kit (BioVision, Milpitas, USA). Phosphatidylserine is normally exposed on the inner leaflet of cellular membrane. In apoptotic cells, it becomes accessible to annexin V.

### Detection of intracellular ROS generation

The generation of intracellular reactive oxygen species was detected by using 2',7'-dichlorodihydrofluorescein diacetate (H_2_DCFDA, LifeTechnologies GmbH, Darmstadt, Germany). H_2_DCFDA is a non-fluorescent probe that diffuses into the cell where its ester bonds are cleaved by intracellular esterases. In the presence of intracellular ROS, H_2_DCFDA become oxidised and is converted to the fluorescent 2',7'-dichlorofluorescein, which is no longer cleavable. Thus, green fluorescence is an indicator of ROS activity. HUVEC were seeded on collagen-coated 12 mm cover slips in 24 well plates with hydrocortisone-free medium and cultivated until confluence. Medium was removed and thermal as well as mechanical damage was induced using the two-ring stamp. Cells were washed once with warm medium and incubated in 1 ml of medium at 37 °C in humidified air containing 5 % CO_2_. 30 min before the selected time points, H_2_DCFDA was added to 10 µM, and incubation continued at 37 °C. Cells were washed twice with medium equilibrated at 37 °C in the dark, and the green fluorescence (ca. 490 nm excitation) was determined using the microscope (Axiovert 200M, Carl Zeiss AG, Oberkochen, Germany). As the ROS generation is induced by light, the microscopic image capture was performed. For quantification, software-assisted image analysis was performed in which the total areas covered by fluorescent cells were compared. To this end, a threshold fluorescence value was defined, binarising the images according to fluorescent and non-fluorescent cells. Cells damaged only mechanically using the unheated stamp served as a reference.

### Immunofluorescence

HUVEC were grown on sterile glass coverslips as described above and thermally affected with the two-ring stamp at 100 °C for 1 sec. As control the stamp was used unheated. After regeneration of the cells for 1, 3, 6 and 24 h, the medium was removed and the cells were fixed with 4 % PFA at RT for 10 min for cleaved caspase-3 and phospho-Hsp 27 staining and with 100 % ice-cold methanol for 10 min for Hsp 70 staining. After fixation, the cells were washed three times with PBS at room temperature and permeabilised with 0,1 % Triton in PBS for 10 min. After washing the cells three times with PBS, they were blocked with culture medium for at least 1 h at room temperature. The coverslips were inverted and placed on 40 µl of diluted primary antibody on parafilm. For incubation over night at 4 °C a wet chamber was used. Cleaved caspase-3 (Asp175) (Cell Signaling, NEB, Frankfurt am Main, Germany) was diluted 1:400, phospho-Hsp 27 (Ser82) and Hsp70 (Cell Signaling, NEB, Frankfurt am Main, Germany) were diluted 1:50 in culture medium. The cells were then washed three times before incubating them with the secondary antibody (Cy3 anti-rabbit, DIANOVA GmbH, Hamburg, Germany) in a dilution of 1:200 in culture medium for 1 h in the dark. When phalloidin staining was performed, cells were incubated with Oregon green 488 phalloidin (Life Technologies GmbH, Darmstadt, Germany) in PBS (0.33 µM) for additional 30 min at room temperature. Cells were washed three times with PBS before and after phalloidin staining. For nuclear staining, DAPI (4',6-diamidino-2-phenylindole) was used at a final concentration of 0,5 µg/ ml. Cells were stained for 10 min, washed with PBS three times and finally the coverslips were mounted on slides using fluorescence mounting medium (Dako, Hamburg). The slides were analysed using the Axiovert 200M microscope (Carl Zeiss AG, Oberkochen, Germany) and images were obtained using the Axiovision 4.8 software (Carl Zeiss AG, Oberkochen, Germany). The fluorescence intensities of the nuclear phospho-Hsp 27 (pHsp 27) and the total of Hsp 70 were determined by a semi-automated computer-based analysis using the ImageJ 1.45 s software (Wayne Rasband, National Institutes of Health, USA). For pHsp 27 analysis, the mean grey value of three cell nuclei in three different areas with each about 50 µm (affected, non affected and the area between non-affected and affected cells) per image was determined and normalised to the mean grey value of the nuclear localisation of cells in the non-affected area. Areas from 150 to 200 µm at the adjacent area after damage were analysed for Hsp 70 expression. For this analysis we did not differentiate between nuclear and cytoplasmatic expression, but determinded the mean grey value of the whole cells.

### Cell adhesion assay

After stamping the HUVEC monolayer, cells were washed once with equilibrated medium at 37 °C and then cultured dependent on the experiment for 1, 3 or 6 h in hydrocortisone-free medium. The medium was removed from the wells and GFP-labelled THP-1 cells or freshly isolated primary PBMC (2.5 x 10^5^ cells/ ml) were added in RPMI 1640 medium to each well for an additional 1 h at 37 °C in 5 % CO_2_. After incubation, cells were washed twice with medium and were immediately examined under a microscope (Axiovert 200M, Carl Zeiss AG, Oberkochen, Germany) using the Axiovision 4.8 software (Carl Zeiss AG, Oberkochen, Germany). As positive control, HUVEC were stimulated with 10 ng/ ml TNF-α (Sigma-Aldrich GmbH, Taufkirchen, Germany) for 6 h as previously described [16]. As another negative control, eGFP-labelled THP-1 or primary PBMC were incubated for 1 h on collagen-coated cover slips without HUVEC. The experiment was performed in triplicate with a minimum of three wells per experiment and treatment. The number of adhered mononuclear cells was determined by a semi-automated computer-based analysis using the ImageJ 1.45 s software (Wayne Rasband, National Institutes of Health, USA). For screening the wells from the left hand side to the right hand side, single images were assembled in a collage by using Adobe Photoshop CS2 (Adobe Systems GmbH, Munich, Germany). Nine different areas of such collages were selected, in which adhered THP-1 were counted and their density was calculated in relation to the area in cm^2^.

### Microscopy and post-processing of images

**Figure 5:**
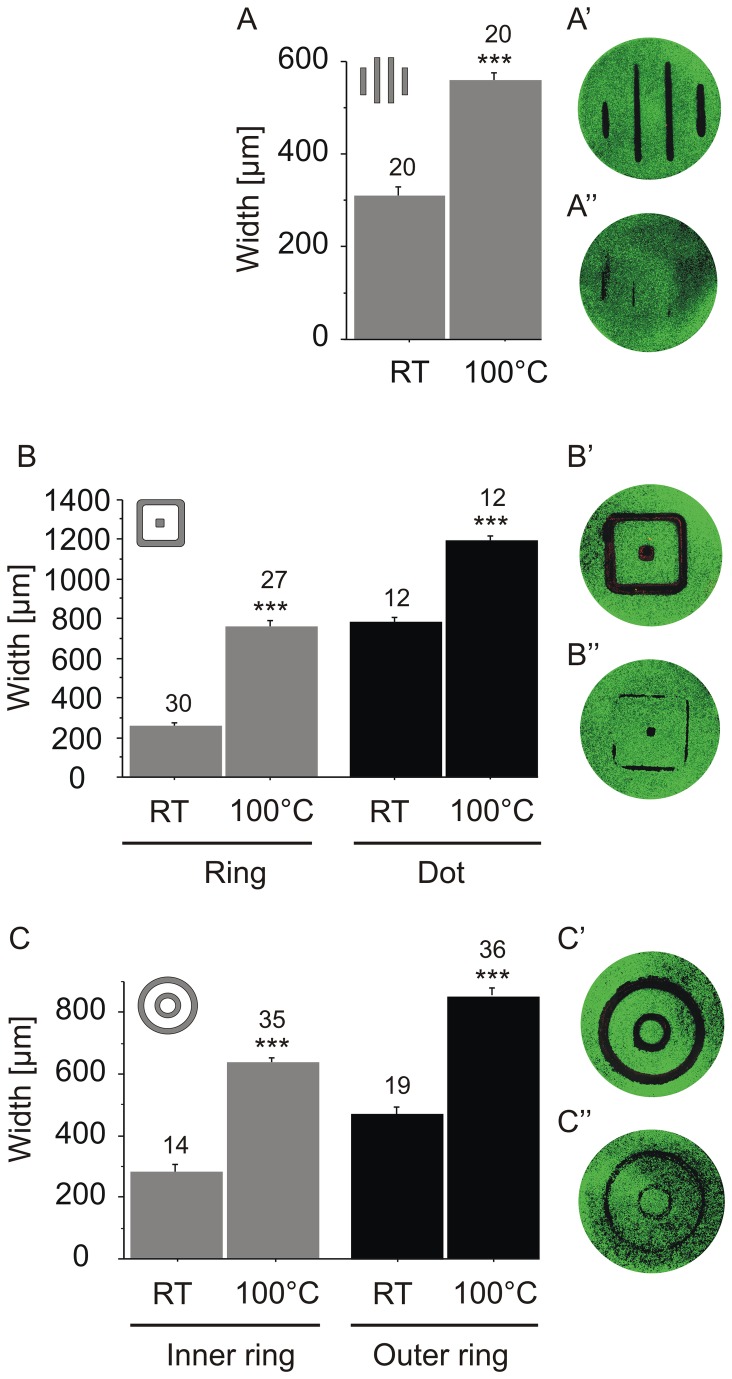
Width of affected areas using three different stamping devices immediately after damage of HUVEC (0h). To investigate the viability of the cells, calcein-propidium iodide staining was used and the width of the damaged areas was measured. A: line-stamp. The width of damaged cells using the unheated stamp (RT) was compared to the width after thermal damage (100 °C). For measurement, the width of the four stripes generated was measured at one position selected at random. A’ and A’’: representative images of 12 mm cover slips with calcein-propidium iodide stained HUVEC as used for the quantification. HUVEC after thermal damage (A’) and after mechanical damage at RT (A’’). This experiment was performed in duplicates whereas the width was measured in 20 independent images B: square-stamp. To discriminate between the width of the outer line and the diameter of the central square, both were measured and quantified in B. The image on the right hand side shows the difference between the thermally damaged HUVEC after calcein-propidium iodide staining (B’) and the unheated stamp control (B’’). C: two-ring stamp. The width of the inner ring and of the outer ring were measured separately for both the thermal damage and the mechanical damage. C’: monolayer of HUVEC after calcein-propidium iodide staining performed immediately after thermal damage (C’) and after mechanical damage (C’’). B and C were performed in triplicates and n>12 independent images were analysed. Numbers on bars indicate the number of images analysed.

**Figure 6:**
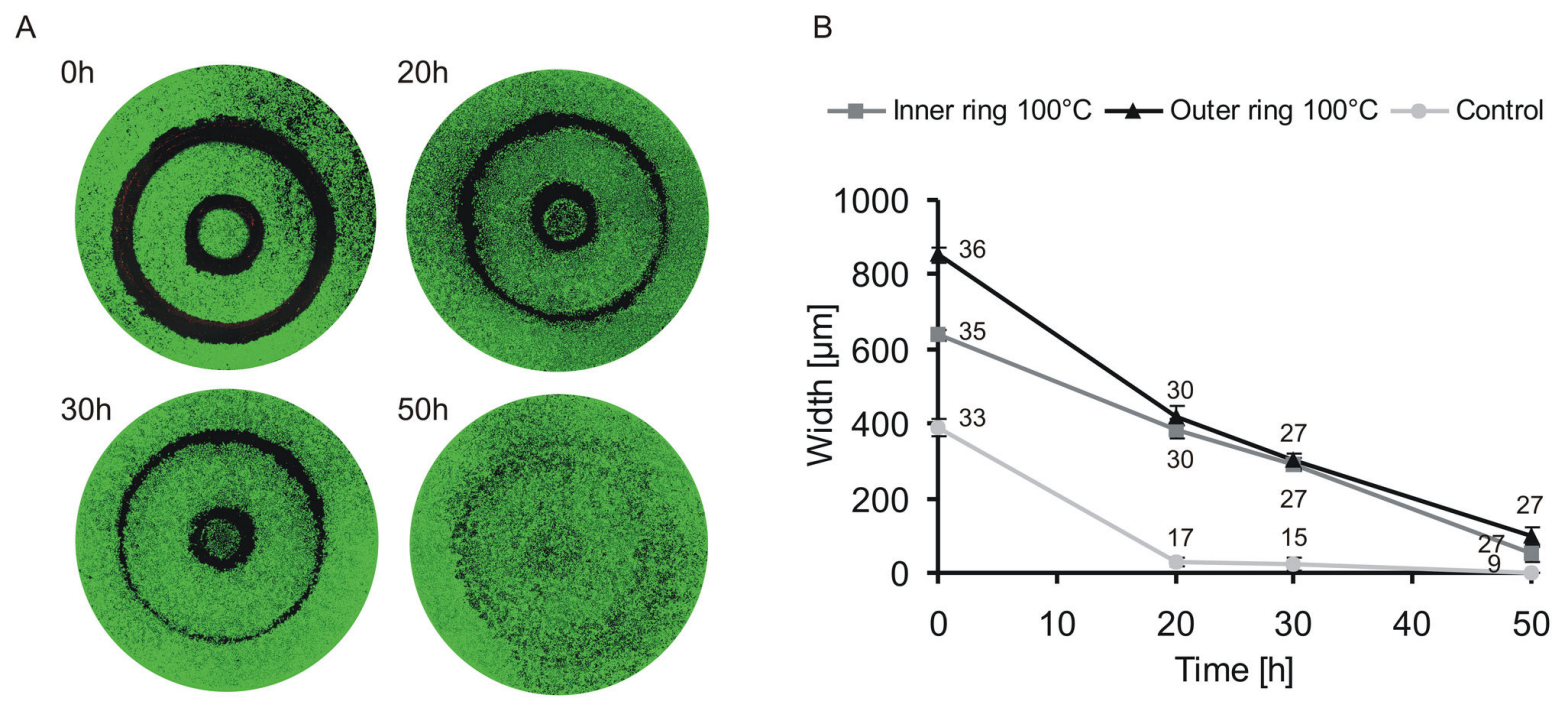
Cell migration into the wounded area after thermal damage. The two-ring stamp was used at 100 °C for 1 sec as described in materials and methods. The migration of HUVEC was analysed after 20, 30 and 50 h, by measuring the width of the affected area after viability staining. Representative images are shown in (A). As control, the affected area was measured immediately after thermal damage (0h). B: quantification of the migrating HUVEC after 0, 20, 30, and 50 h. We differentiated the migration into the inner (■) and outer ring (▲) after thermal damage but did not differentiate after mechanical damage (control ●). For analysis of migration after mechanical damage with the unheated stamp, we pooled the widths of the two rings, because HUVEC already formed an almost confluent monolayer after 20 h. However, after 50 h, the thermally damaged HUVEC formed a monolayer as well, although with a reduced degree of confluency (viability staining (A) 50 h). The experiment was performed in triplicates with a minimum of three wells per experiment and treatment.

All samples were examined under an Axiovert 200M microscope (Carl Zeiss AG, Oberkochen, Germany) and images were captured using the Axiovision 4.8 software (Carl Zeiss AG, Oberkochen, Germany). To obtain an overview of the entire stamping imprints on the 12 mm cover slips, detailed images were captured and combined into a collage using Adobe Photoshop CS2 (Adobe Systems GmbH, Munich, Germany). Image contrast was adjusted to better visualise the profile of the stamping devices (Figures 5 and 6). For all other images, no post-processing was performed.

### Statistical analysis

The data was analysed by using STATVIEW software (SAS-institute Inc, Heidelberg, Germany). ANOVA analysis was carried out, and p values of <0.05 are referred to as *, <0.01 as ** and <0.001 as ***. Error bars represent standard errors of the mean and all experiments were performed at least three times independently, and 2-4 wells were analysed per treatment.

## Results

### The new *in vitro* model generates geometrically highly reproducible damage zones

We tested the stamping devices with three different geometries on primary HUVEC monolayer cell cultures: a four line stamp, a square stamp and a circular stamp (see materials and methods). To test for the reproducibility of the width of the damaged area, we performed a viability staining immediately after thermal damage at 100 °C. The numbers above the bars indicate the number of images quantified. As a control, we stamped cells with the unheated stamp at RT. Figure 5 shows the results of the different model systems using calcein and propidium iodide stained HUVEC. In Figure 5 A we tested the line-stamp, the geometry of which is most similar to the geometry of the damage caused in a scratch assay. As expected, the area damaged, as indicated by lack of calcein stain, was larger after thermal damage than after mechanical damage (Figure 5 A’, A’’). However, the lengths of the two outer thermal damage zones were not identical. Additionally, the footprint of the unheated stamp was irregular (Figure 5 A’’). For analysis, only the width of the two centre lines of the unheated stamp control in A was considered. Still, we could determine a highly significant difference in the width of thermally damaged zone versus the mechanically damaged zone in the HUVEC monolayer (Figure 5 A). In Figure 5 B we tested the square-shaped stamp. For quantification we differentiated the outer line and the dot in the centre (Figure 5 B). The width after thermal damage compared to mechanical damage was highly reproducible in both cases. Figure 5 B’ shows the monolayer after thermal damage with the square-stamp, and Figure 5 B’’ illustrates the footprint of the stamp control. Note that the corners of the footprint of mechanically damaged HUVEC showed inhomogeneous widths (Figure 5 B’’). In Figure 5 C we tested the two-ring stamp geometry. The difference in the width of the damaged areas after mechanical damage versus after thermal damage of the outer ring and the inner ring was highly reproducible. The appearance of the thermally damaged area (Figure 5 C’) as well as of the mechanically damaged area (Figure 5 C’’) were homogeneous. Consequently, for all further experiments we used the two-ring stamp geometry.

### The wound area is closed by the migration of unaffected HUVEC

For the establishment of a suitable *in vitro* model of thermal damage it was necessary to investigate whether unaffected HUVEC are able to migrate into the wounded area. If all cells had been affected, no further investigation of wound healing would have been possible. To examine the ability of the unaffected cells to proliferate and to migrate, we performed migration assays monitoring the closure of wounds. On the left panel of Figure 6 A, representative images of calcein-stained HUVEC cells are shown at four different time points after thermal damage using the two-ring stamp. For each time point (0, 20, 30 and 50 h) after thermal damage, we quantified the width of the wounded area in Figure 6 B. We noticed that the migration of the cells into the outer and the inner ring followed a different pattern. Therefore we differentiated between the migration into the outer (▲) and the inner ring (■). The graph shows that in both cases HUVEC were able to migrate into the affected areas. After 50 h, the wounds were almost closed, but in the formerly damaged zones the cell layer was not as confluent as in the unaffected areas. We also compared the migration time of thermally damaged HUVEC to HUVEC damaged mechanically using the unheated two-ring stamp. As already shown in Figure 6, the width of the damaged area is approx. half that of the area after thermal damage. Therefore, the closure of the monolayer was faster than that of the thermally damaged cells (Figure 6 B). For quantification of the migration of the control (●) we did not differentiate between the width of inner and other ring, as after 20 h the monolayer was already confluent in both cases.

### Thermal damage results in consecutive zones of differently affected HUVEC

**Figure 7:**
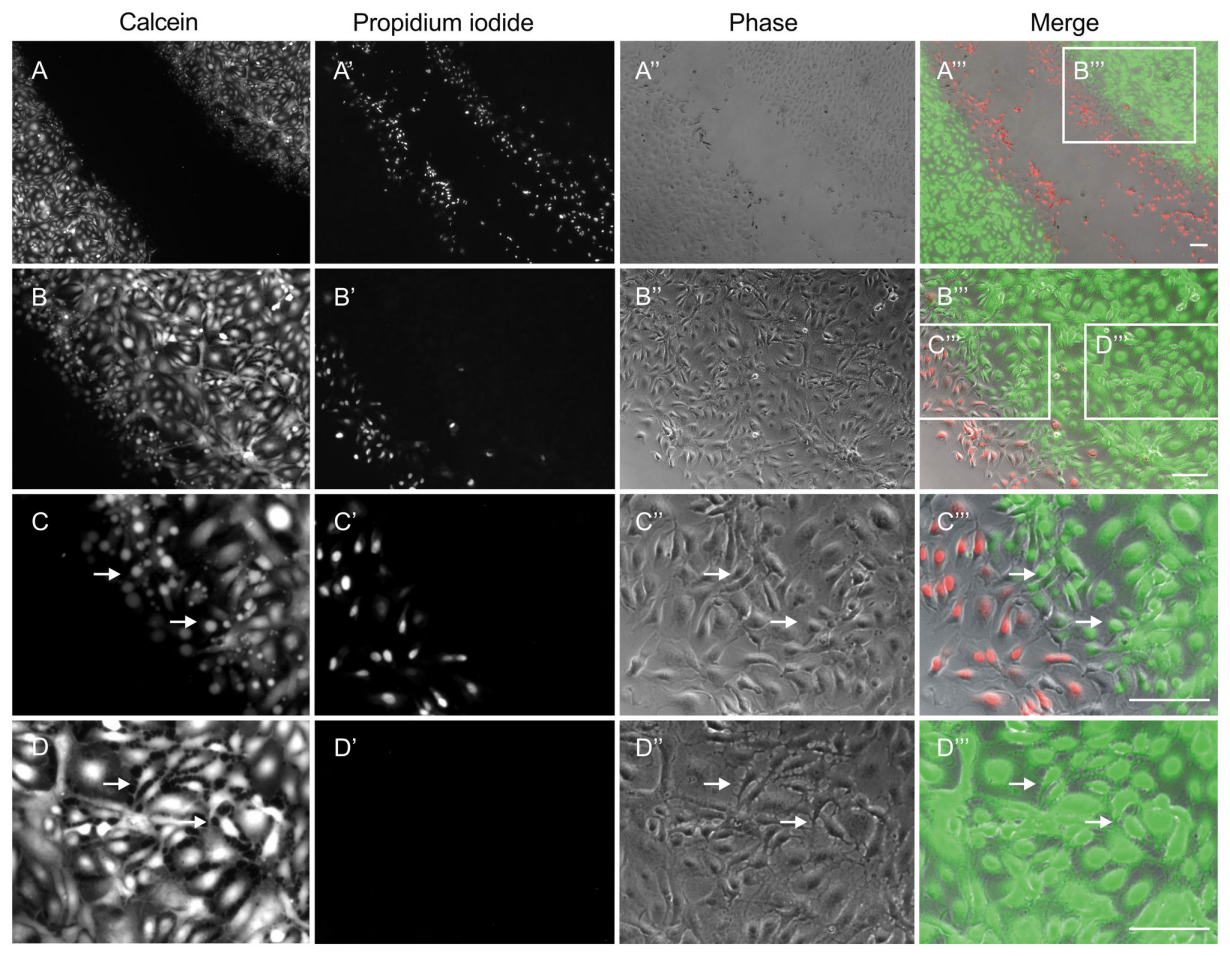
Viability staining of HUVEC immediately after thermal damage (100 °C). The two-ring stamp was used for 1 sec as described in materials and methods. The green fluorescence of the calcein indicates vital cells (A-D), whereas the red fluorescence of propidium iodide indicates dead cells (A’-D’); due to the staining procedure, the time that elapsed between damage and image acquisition was between 30-60 min. HUVEC in phase contrast are illustrated in A’’-D’’, mergers of the different images are shown in A’’’-D’’’. A representative section of the damaged area is shown in (A-A’’’). It highlights the inner ring of the two-ring stamp, but a similar pattern as described in the following was also detectable in the affected area of the outer ring (data not shown). B-B’’’: enlargement of the affected area. The affected area in detail showed different phenotypic effects on different cells. C-C’’’: in between dead and vital cells, dying cells were present. Farther away from the damaged cells, two to five lines of HUVEC were losing their cell-cell contacts (D-D’’’). Both cells already dying as well as cells losing their cell-cell contacts are indicated with arrows (Scale bars: 100 µm).

Immediately after thermal damage, differently affected zones were discernible in the HUVEC monolayer, presumably due to heat radiation and/or heat conduction. We could not only differentiate between vital and dead cells but also distinguish apoptotic cells and cells losing their cell-cell contacts. Figure 7 shows a representative section of the inner ring after thermal damage with 100 °C for 1 sec and subsequent enlargements thereof. Due to the staining procedure, these pictures were taken approximately 30 min after thermal damage. A similar pattern of cellular damage zones after thermal damage occurred at the outer ring (data not shown). The affected area of the HUVEC was determined using viability staining employing Calcein-AM for staining live cells and propidium iodide for staining dead cell as described in materials and methods. An overview of the section under study is shown in Figure 7 A-A’’’. Viable cells are shown in A, a stretch of dead cells is discernible in A’ a phase contrast image is given in A’’and a merger is given in A’’’. The right border of the inner ring’s footprint featuring dead cells and vital cells is enlarged in Figure 7 B, B-B’’’. Two further enlargements of Figure 7 B’’’ are given in Figures 7 C and 7 D: in Figure 7 C, C-C’’’ the border of dead and living HUVEC is shown in more detail. Arrows in C, C’’ and C’’’ point at cytoplasmic blebs [17] in cells that were in the immediate vicinity of dead cells. These cells are still alive because they were positively stained with calcein, but these blebs or ‘popcorn cytolysis’ is indicative of dying cells [18].Vital cells further away from the damage zone display a morphology typical for HUVEC (Figure 7 D-D’’’). However, some of these cells have apparently lost their cell-cell contacts (marked with arrows in D, D’’ and D’’’). Therefore, the damage zone extends about tree to five lines of cells into the area of vital HUVEC. Using live cell imaging, we could see that most of those cells which have lost their cell-cell contacts died after a few hours (data not shown). We conclude that different stages of vital to dead cells are present in our model system. In contrast to the established scratch assay, in our model the cell debris remains, analogous to the situation *in vivo*.

### Cell stress due to heat results in local cell death

**Figure 8:**
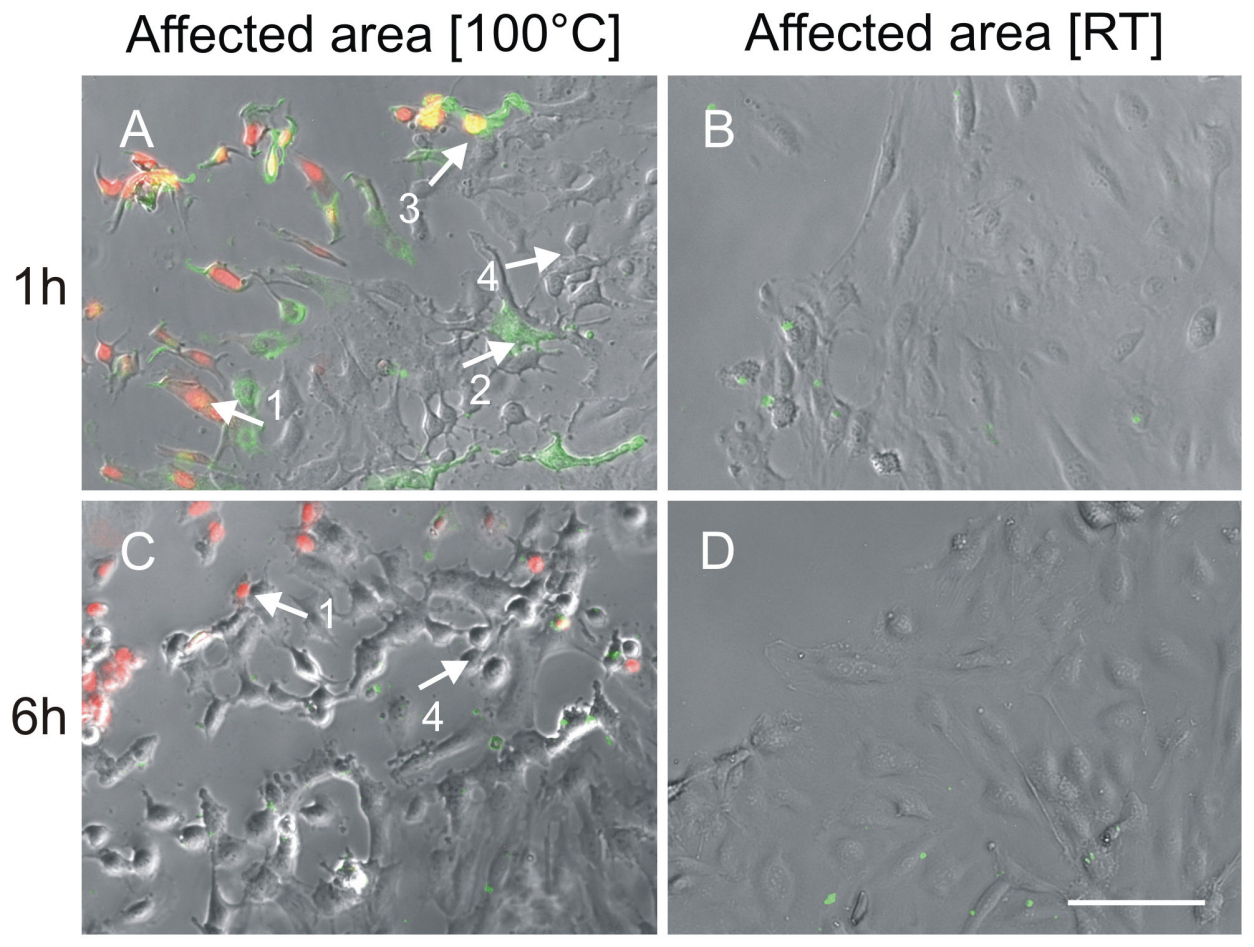
Necrotic and apoptotic cells after thermal damage. HUVEC were damaged using the two-ring stamp at 100 °C for 1 sec as described in material and methods and stained for necrotic cells (1: propidium iodide, red) as well as early apoptotic cells (2: Annexin V, green). One hour after thermal damage both necrotic as well as early apoptotic cells are present (A). In contrast, no cell death is seen after mechanical damage (B). 3: co-localisation, yellow: late apoptotic cells or necrotic cells. 4: vital but rounded cells at the adjacent area after thermal damage are typical for this region and are distinguishable in the phase contrast due to lack of staining. C: HUVEC at the adjacent area 6 h after thermal damage. Necrotic but no apoptotic cells are seen. D: No cell death is seen at 6 h after mechanical damage. Scale bar: 100 µm.

**Figure 9:**
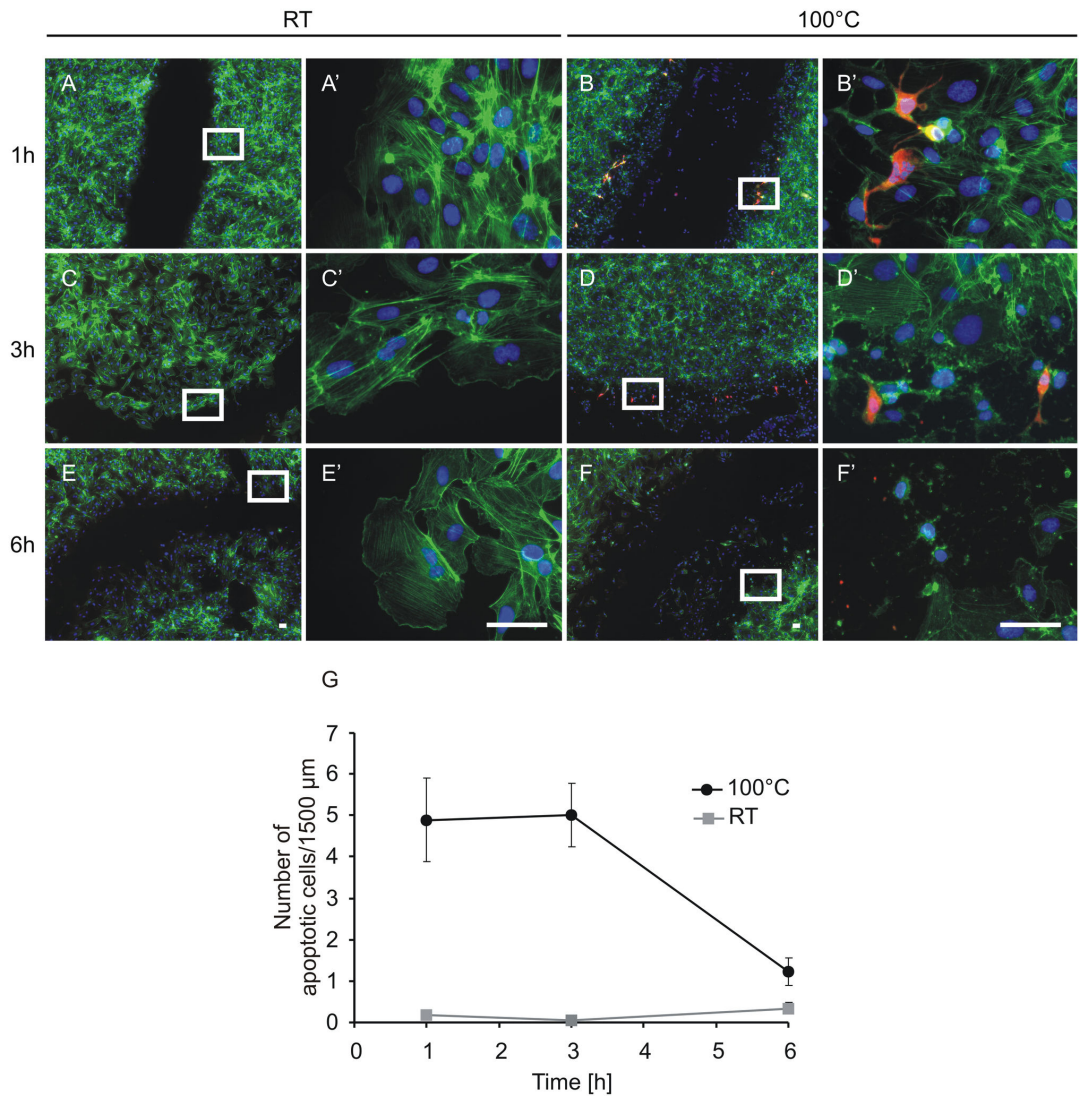
Caspase 3 activity indicates apoptotic cells at the adjacent area after thermal damage. A-F’: Representative images of the immunofluorescence staining of HUVEC 1, 3 and 6 h after mechanical (A-A’, C-C’, E-E’) and thermal damage using the two-ring stamp at 100 °C for 1 sec (B-B’, D-D’, F-F’). Green: actin cytoskeleton (Phalloidin). This staining was used additionally in order to better discriminate live cells from apoptotic and dead cells, blue: nuclei (DAPI), red: active caspase 3 (Cy3). Scale bars: 50 µm. A’, B’, C’, D’ E’ and F’ represent the selected areas of A, B, C, D E and F in detail. G: quantitation of the cells with Caspase 3 activity that are shown in A-F’. The number of apoptotic cells was determined by counting the red fluorescent cells along a line (1500 µm) parallel to the edge of the damaged zone. The experiment was performed in triplicates, n= 18 lines were analysed. One to three hours after thermal damage, we counted approximately 5 cells with caspase 3 activity in a line of 1500 µm. The number of apoptotic cells decreased over time so that only 1-2 apoptotic cells were present in this area after 6 h. There was no difference in the number of apoptotic cells after thermal damage detectable.

Probably, cells die immediately after being exposed to pressure and due to the extreme heat at the adjacent area. To clarify if there are only necrotic cells or also apoptotic cells after thermal damage, we performed an Annexin V- propidium iodide staining one hour and six hours after damage. Detailed pictures of the adjacent area after thermal and mechanical damage are shown in Figure 8. In the area exposed to both pressure and heat, we detected necrotic cells after one hour as well as after six hours. Apoptotic cells were present at the adjacent area one hour after damage whereas we could not detect apoptotic cells after six hours any more. Furthermore, the colocalisation of green and red fluorescence indicates late apoptotic or necrotic cells (Figure 8 A). Additionally, these pictures show unstained HUVEC that have undergone a morphological change. Instead of the typical brick-like shape, they exhibit a more rounded morphology (Figure A, C, arrow 4), indicating that not all cells at the adjacent areas are on the way to cell death at this time. In contrast, neither necrotic nor apoptotic cells were detectable after mechanical damage only (Figure 8 B, D). To clarify the presence and absence of apoptotic cells, we performed an additional staining for cleaved caspase-3 one, three, and six hours after mechanical and thermal damage (Figure 9). Effector caspases (caspase-3 and 7) are activated by initiator caspases through the intrinsic as well as through the extrinsic pathway during apoptosis [19]. Thus, activated caspase-3 is a useful marker for apoptotic cells. In accordance with the Annexin V staining, we detected cells with cleaved caspase-3 only in the area adjacent to thermal damage, one hour and three hours after inflicting the damage (Figure 8). In contrast, no caspase-3 activity was detected in cells after mechanical damage using the same stamping device.We have added phalloidin staining (labelling the actin cytoskeleton) in order to better discriminate live cells from apoptotic and death cells. Six hours after thermal damage fewer apoptotic cells were present.

### Thermal damage induces reactive oxygen species (ROS) in HUVEC

**Figure 10:**
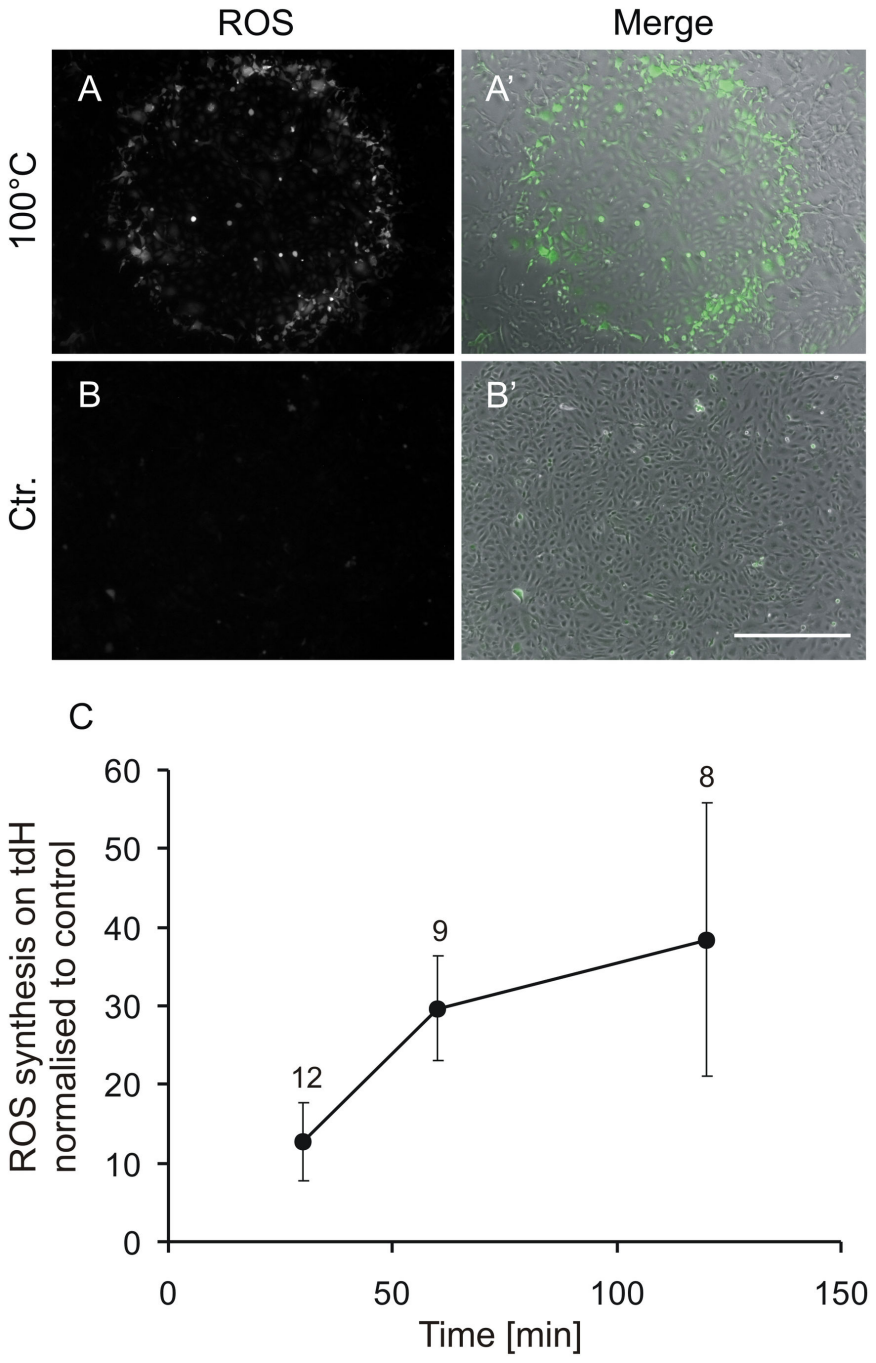
ROS synthesis at the area adjacent to thermally damaged HUVEC (tdH). To detect ROS, the fluorochrome H_2_DCFDA was used: the intensity of the green fluorescent signal of the region limited by the inner ring was analysed. Representative images of ROS synthesis 30 min after thermal (A, A’) and after mechanical (B, B’) damage. A and B, green fluorescent ROS, merge of HUVEC (phase contrast) and green fluorescent ROS (‘). No ROS synthesis was detected after using the unheated stamp (B). Scale bar 500µm. C: quantification of the ROS synthesis normalised to the control. After 30 min, a 10-fold increase after thermal damage in HUVEC was observed compared to the unheated stamp control, and a 30-fold increase was observed after 1 h. Two hours after thermal damage the signal varied substantially, as is indicated by the error bar. The experiment was performed in triplicates, n>8 images were analysed.

As it has been previously demonstrated that ROS synthesis is a response to heat stress [20], we investigated whether vital HUVEC in the vicinity of thermally damaged cells were likewise stimulated. As we had already observed that HUVEC situated in the inner ring seemed to be more affected by the heat (see Figure 10), we selected this central area (Figure 10 C, area 5) for analysis. We used green fluorescent H_2_DCFDA to visualise ROS synthesis as described in materials and methods. The ROS synthesis in the central area 30 min after thermal damage is illustrated in Figure 10 A and A’ (merge of HUVEC in phase contrast and green fluorescence indicating ROS). In contrast to the thermally affected HUVEC, cells which were damaged mechanically showed no ROS synthesis (Figure 10 B, B’). For quantification, we normalised the ROS synthesis after thermal damage to the control after 30 min, 1 h and 2 h. We could observe a 10-fold increase of ROS after 30 min and about a 30-fold increase after one hour (Figure 10 C). Two hours after thermal damage, the fluorescence signals became very variable. To summarise, ROS is synthesised shortly after thermal damage underscoring our assumption that HUVEC in our new *in vitro* model are not only damaged, but that they react to the exposure of heat with stress, attracting mononuclear cells to the site of damage.

### Not only cell death but also cell rescue is a reaction of thermally affected HUVEC

**Figure 11:**
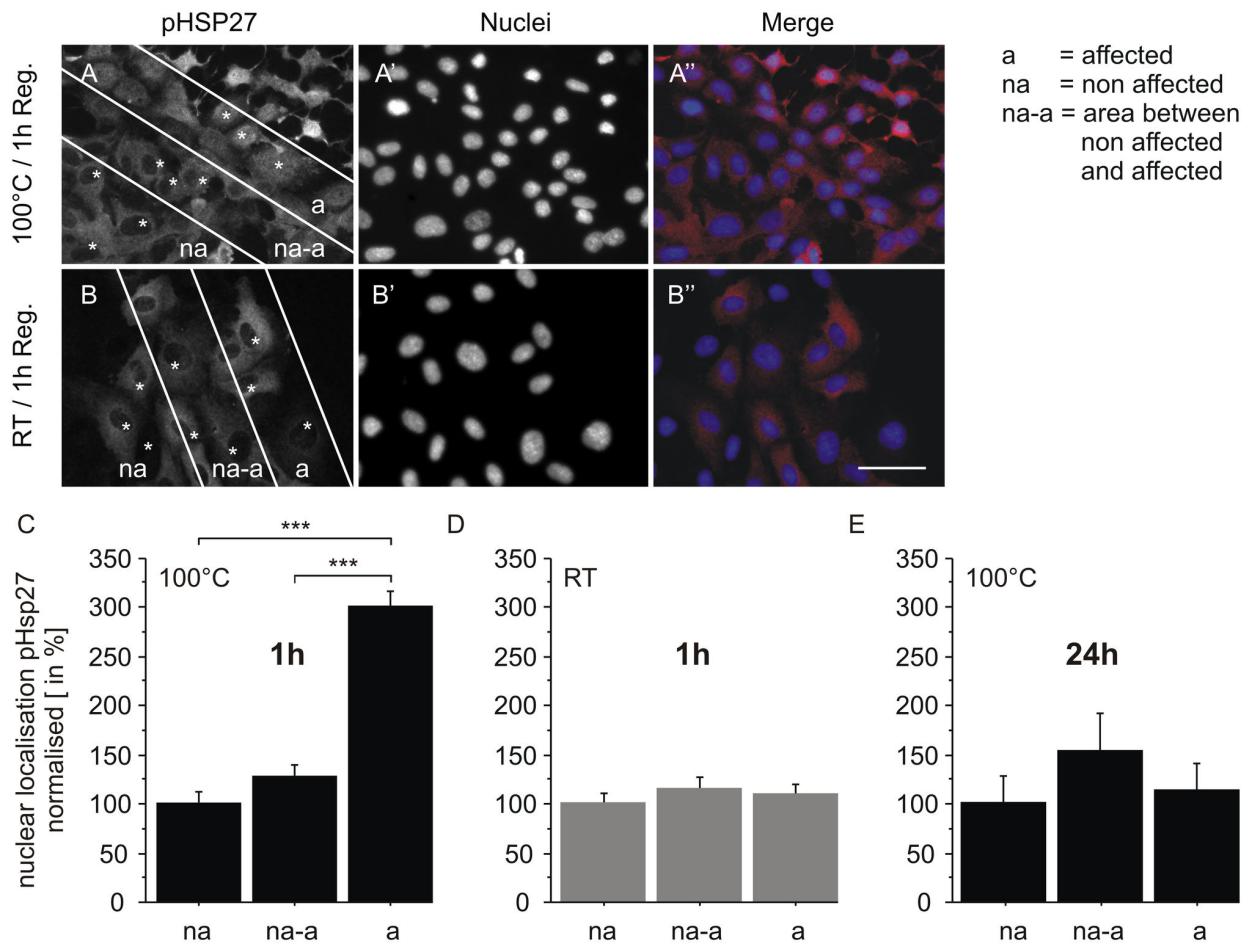
Phospho-Hsp 27 expression in HUVEC at the adjacent area after thermal damage. A-A’’: representative images of phosho-Hsp 27 (pHsp 27) expression 1 h after thermal damage with 100 °C for 1sec, and after mechanical damage at RT (B-B’’): first column – pHsp27 (Cy3), second column- nuclei (DAPI), third column – merge. Three cells (labelled with *) in each zone (50 µm wide) were used for analysis (A and B). Scale bar: 50 µm. Quantification of pHsp 27 one hour after thermal damage is shown in C and after mechanical damage in D. HUVEC at the adjacent area after thermal damage showed a highly significant increase of nuclear localisation of pHsp 27 one hour after thermal damage (C). In contrast, no nuclear localisation of pHsp 27 was found after mechanical damage (D). Aditionally, no nuclear pHsp 27 expression was seen after 24 h (E). The experiment was performed in triplicates and n=33 independent images were analysed.

**Figure 12:**
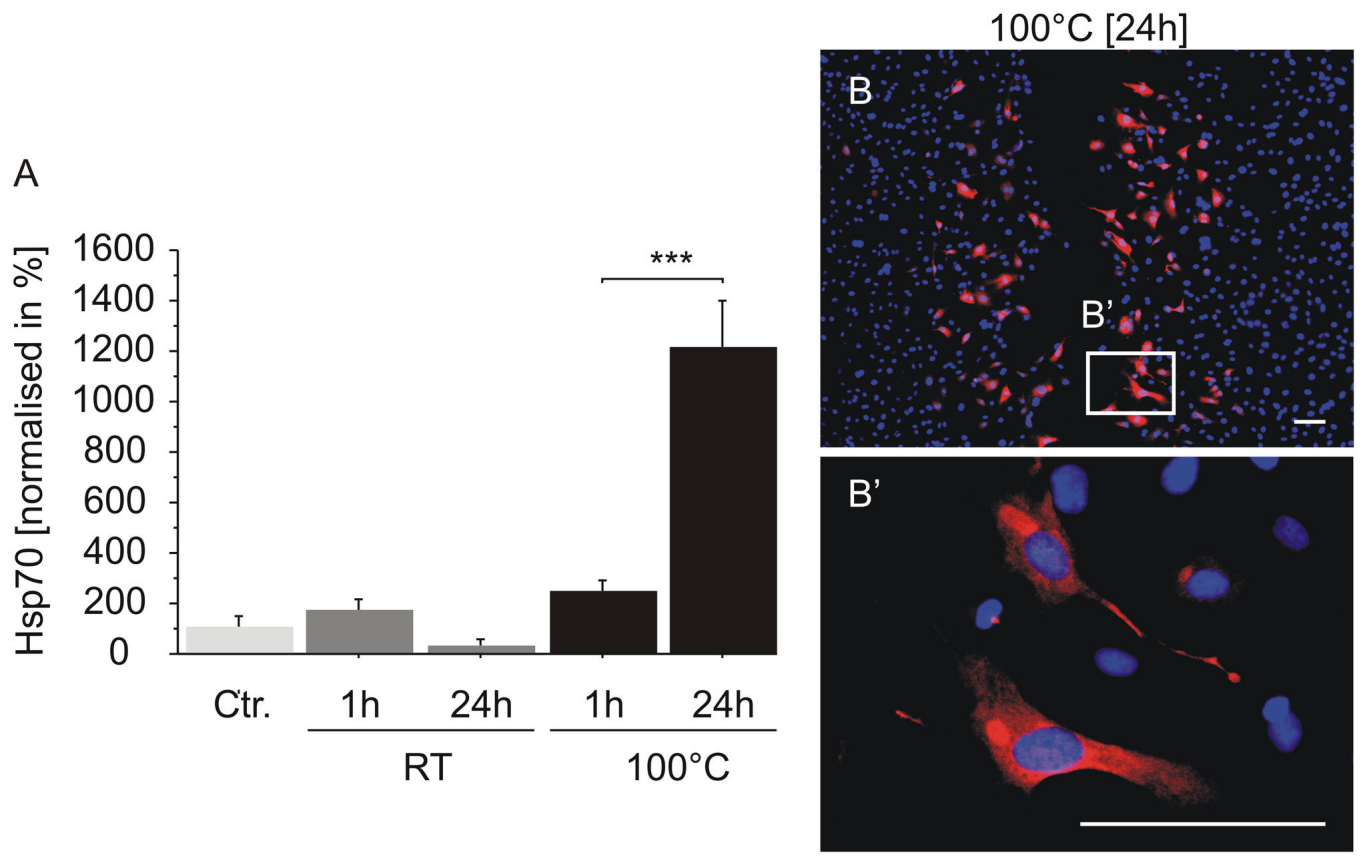
Hsp 70 expression in HUVEC at the adjacent area after thermal damage. A: quantification of Hsp 70 expression in unaffected HUVEC (Ctr.), in mechanically affected HUVEC (RT) and in thermally affected (100 °C) HUVEC using the two-ring stamp. Hsp 70 expression was analysed in an area of 150-200 µm width adjacent to the “wound” (B). B: representative image of Hsp 70 expressing cells 24 h after thermal damage (stained with Cy 3) close to the “wound”. Nuclei were stained with DAPI. B’: enlargement of B. Scale bars: 100 µm. The experiment was performed in triplicates and n=24 (Ctr., 24 h) and n=27 (1 h) independent images were analysed.

When cells are exposed to elevated temperatures they respond with the synthesis of heat shock proteins [21]. Apart from functions, heat shock proteins act as antioxidants to reduce ROS synthesis and act as suppressors for different kinds of cell death [22]. Since we could prove that cell death and ROS synthesis are locally confined at the adjacent area after thermal damage using the two-ring stamp, we supposed that some of the affected cells should be protected from cell death as well. To investigate this we performed an imunofluorescence staining to detect whether heat shock proteins are activated or not. We have focussed on the two heat shock proteins phospho-heat shock protein 27 (pHsp 27) and Hsp70, for which detailed kinetic studies have already been published. According to these data, the activation of Hsp27 by phosphorylation peaks approx. 1 h after heat shock and declines thereafter [23], whereas Hsp 70 was not detectable after 1 h, but after 24 h following heat shock [24]. Consequently, HUVEC were stained for pHsp 27 (see Figure 11) and Hsp 70 (Figure 12) 1 h and 24 h after thermal and mechanical damage. Representative images of pHsp 27 expression 1 h after thermal damage are given in Figure 11 A-A’’. B-B’’ shows the pHsp 27 expression 1 h after mechanical damage. Three different areas (50 µm in width) were chosen for analysis. The first area close to the “wound” with the affected cells (a) an additional area with non affected cells (na), and the area in between (na-a). In contrast to the mechanically damaged cells (Fig. 11 D), thermally affected cells showed a highly significant increase of nuclear localisation of pHsp 27 (see Fig. 11 C, a). We could not detect nuclear localisation in the other areas after thermal damage (Fig. 11 C na, na-a). Twenty-four hours after thermal damage, the HUVEC showed a re-localisation of pHsp 27 into the cytoplasm, therefore we could not detect nuclear localisation anymore (Fig. 11 E). Figure 12 indicates the Hsp 70 expression of HUVEC after they were treated similarly to Figure 11. Because there is no difference in the localisation of Hsp 70, we did not differentiate between different areas as was done for analysis of pHsp 27 expression. Here, merely just analysed the affected cells at the adjacent area of damage. In contrast to pHsp 27, Hsp 70 was not detectable 1 h after thermal damage (Fig. 12 A). As expected, there was no expression in the untreated control, nor 1 h after mechanical damage neither, whereas 24 h after the cells were thermally affected with the two ring stamp, they showed a highly significant increase of Hsp 70 in the cytoplasm as well as in the nucleus (Fig. 12 A). A representative image of the Hsp 70 expression 24 h after thermal damage at the adjacent area of the “wound” is illustrated in Figure 12 B. An enlargement is shown in 12 B’. Due to the presence of heat shock proteins at different time points we suppose that these affected cells are protected from cell death, but also lead to activate stress signalling pathways, which underscores the suitability of our model for further investigations on thermally effects on monolayer cell cultures.

### Thermal but not mechanical damage caused adhesion of monocytes (THP-1) on HUVEC in a time dependent manner

**Figure 13:**
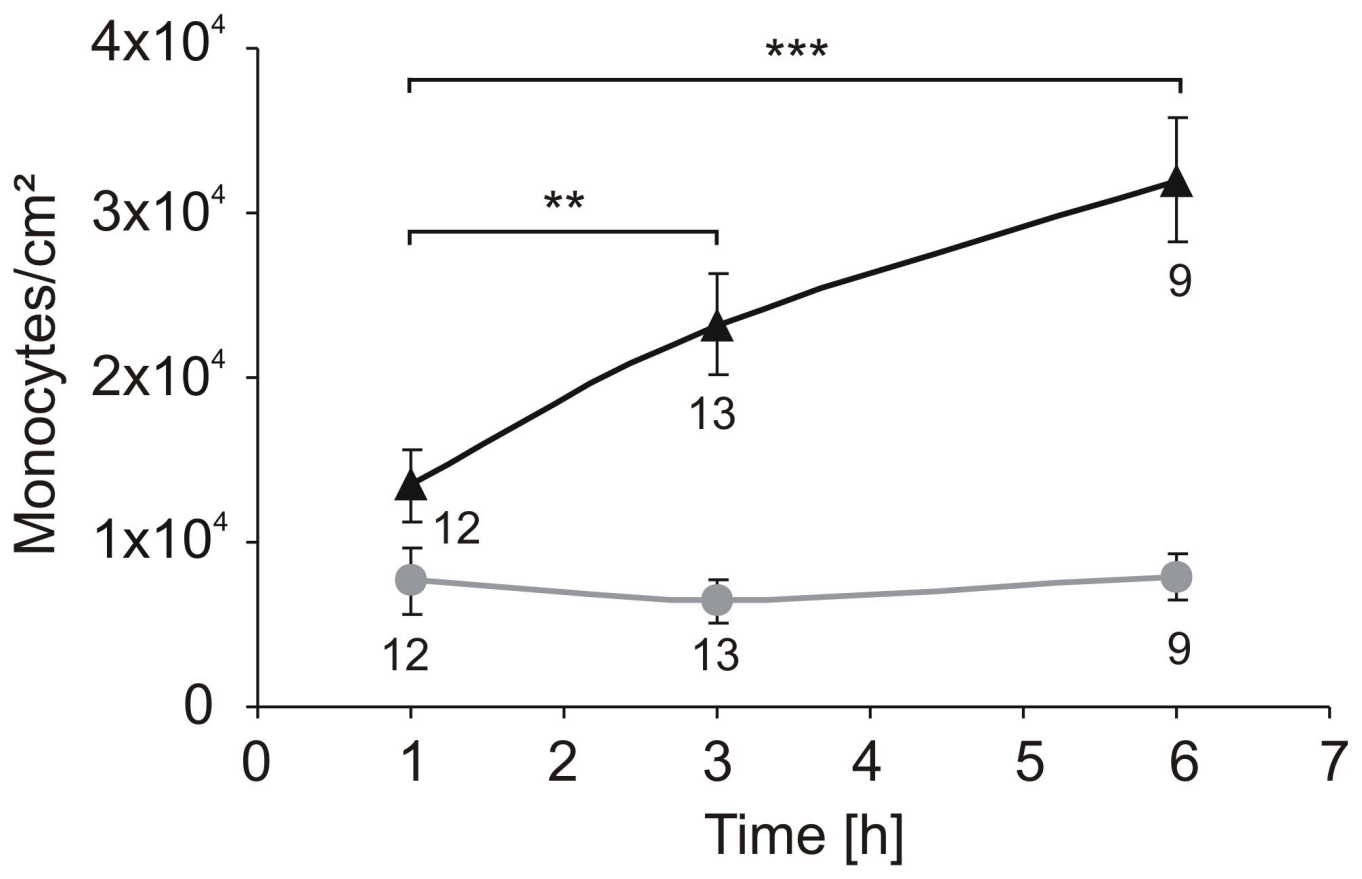
Time dependent adhesion of THP-1 on thermally and mechanically damaged HUVEC. THP-1 adhesion 1, 3 and 6 hours after thermal damage (▲) compared to mechanical damage (●). No difference in THP-1 adhesion after mechanical damage was detectable whereas a significant increase in the number of attached THP-1 cells after 6 h was found. The experiment was performed in triplicates. The labels below the curves indicate the number of pictures taken for analysis.

The adherence of leukocytes, neutrophils and monocytes to the endothelium is a prerequisite for migration to the site of inflammation [12], therefore adherence is a hallmark for an ongoing process of wound healing. To investigate whether monocytes are able to recognise the wounded area in our model, we performed a co-culture of HUVEC and monocytes (THP-1). The HUVEC monolayer was damaged with the two-ring stamp heated at 100 °C and compared to the unheated stamp at room temperature (RT) as previously described in materials and methods. After 0 h, 2 h, and 5 h of incubation, THP-1 cells were added, and the adhered THP-1 cells were counted after an additional hour incubation, using fluorescence microscopy. The outcome of the THP-1 adhesion experiment is illustrated in Figure 13. No difference in THP-1 cell adhesion after mechanical damage (●) was detectable. In contrast, a significant increase of the number of adherent monocytes was detectable 6 h after thermal damage (▲), indicative of processes in the adjacent living cells that mediate such adherence.

### Adhesion of mononuclear cells (THP-1 and PBMC) 6 h after thermal damage is comparable to TNF-α-mediated adhesion

**Figure 14:**
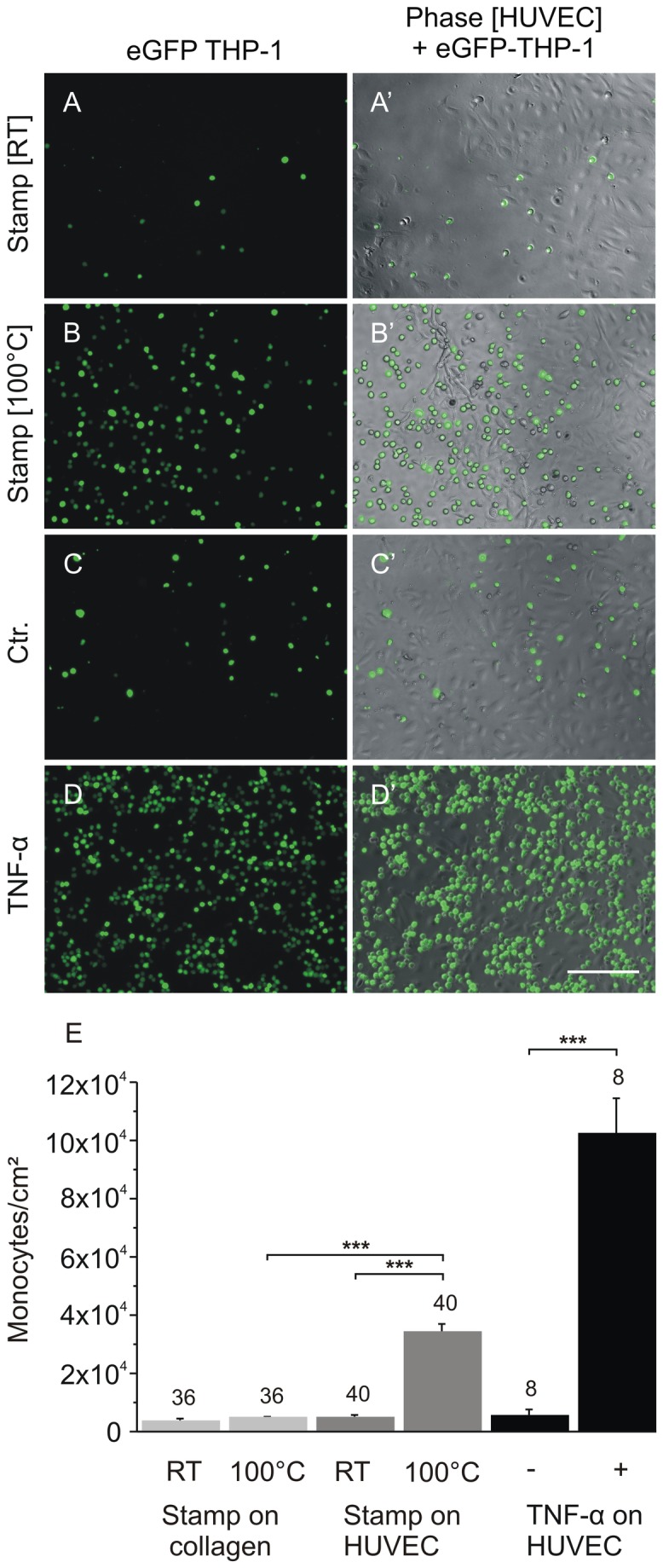
Adhesion of THP-1 on HUVEC 6 h after damage compared to untreated and TNF-α stimulated HUVEC. A, A’-D, D’: representative images of adhered eGFP-labelled monocytes (THP-1). Left images (A-D) THP-1 (eGFP) only. Right images (A’-D’): corresponding merge image with HUVEC (phase contrast) and adherent THP-1 cells. A, A’: unheated stamp control (mechanical damage), B, B’: fluorescence area adjacent to thermal damage, C, C’: untreated HUVEC as control, D, D’: TNF-α-treated (10 ng/ml) HUVEC. Scale bar: 500µm.E: quantification of adhered THP-1 cells per cm^2^. The strongest increase in THP-1 number observed after treatment of HUVEC with TNF-α. The analysis showed a significant increase compared to the number of adhered THP-1 cells on untreated HUVEC. Although the number of THP-1 cells after thermal damage of HUVEC is lower compared to that on TNF-α treated cells, it is highly significant compared to the number of adherent THP-1 cells on mechanically damaged cells. For this analysis we pooled the counted THP-1 cells of the areas 2, 4, 6, and 8 shown in Figure 16 C for thermal damage and the areas 1, 3, 7, and 9 for mechanical damage to get an overview of the whole affected zones. As another negative control, the adhesion of THP-1 cells on collagen-coated cover slips was used to illustrate that THP-1 cells do not simply adhere on thermally compromised collagen. The experiment was performed in triplicates. THP-1 on TNF-α treated cells were counted in n=8 images, and n>36 after thermal and mechanical damage.

**Figure 15:**
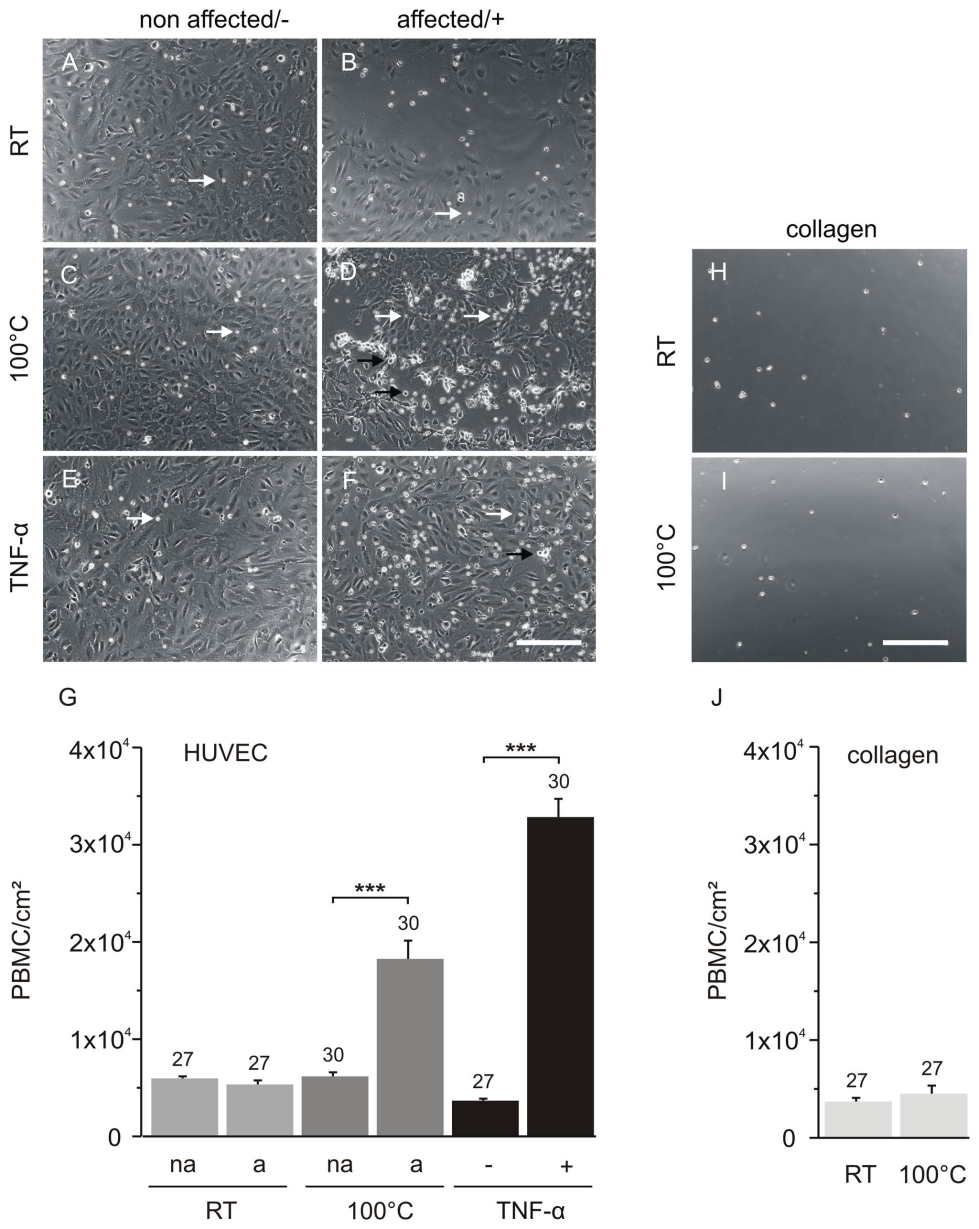
Adhesion of PBMC on HUVEC 6 h after damage compared to untreated and TNF-α stimulated HUVEC. A-F: representative images of adhered PBMC on HUVEC. Left images (A, C, E) adhered PBMC on non-affected HUVEC. Right images (B, D, F): adhered PBMC on affected HUVEC. A, B: unheated stamp control (mechanical damage), C, D: thermal damage, E: untreated HUVEC as control, F: TNF-α-treated (10 ng/ml) HUVEC. Small rounded PBMC that were counted are indicated with white arrows. Larger round cells that cannot be identified clearly as either larger PBMC or dead round HUVEC, were not counted for this analysis and are indicated by black arrows in D and F. G: quantification of adhered PBMC cells per cm^2^. Na = non affected, a = affected, (-) = untreated HUVEC, (+) = treated HUVEC with TNF-α, RT = mechanical damage at room temperature, 100°C = thermally affected (100°C) HUVEC using the two-ring stamp. The analysis showed a significant increase after treatment of HUVEC with TNF-α as well as after thermal damage compared to the corresponding negative control and compared to the number of adhered PBMC on mechanically damaged cells. As another negative control, the adhesion of PBMC on collagen-coated cover slips was used to illustrate that PBMC do not simply adhere on thermally compromised collagen. H: representative image of adhered PBMC on collagen after mechanical damage, I: adhered PBMC on thermal damaged collagen, J: quantification of adhered PBMC cells per cm^2^ on collagen. There is no increase of adhered PBMC after affecting simply the collagen-coated coverslips. The experiments were performed in triplicates, but for the adhesion experiments on collagen which were performed in duplicates. PBMC on HUVEC were counted in n > 27 images, and n = 21 images were counted for adhesion on collagen. Scale bars: 200 µm.

The proinflammatory cytokines interleukin-1 (IL-1) and tumour necrosis factor α (TNF-α) as well as bacterial lipopolysaccharide are known to stimulate endothelial cells to direct lymphocyte adhesion [25]. Thus, we wanted to analyse whether the adhesion invoked by thermal damage is comparable to the extent of adhesion mediated by the administration of TNF-α. As shown in Figure 14 we first analysed the adhesion of THP-1 cells on HUVEC. As shown in Figure 14 E, the number of adherent THP-1 cells in the areas affected by thermal damage using the two-ring stamp reached aaprox. 40 % of the number of cells that adhered after stimulating the HUVEC with TNF-α. Thus, although the THP-1 cells adherence is lower, it is in a comparable order of magnitude. To exclude that THP-1 cells merely adhere to thermally compromised collagen, i.e. independent of thermally affected HUVEC, we repeated the adhesion assay on thermally or mechanically stamped collagen-coated cover slips, in the absence of HUVEC. As shown in Figure 14 E, neither the thermal nor the mechanical damage of collagen had any influence on the number of THP-1 cells becoming adherent, in line with the findings above, which indicated a necessary contribution of the living cells nearby. Figure 14 A, A’ – D, D’ illustrates the THP-1 adhesion on differently treated HUVEC. The left column (A-D) shows the green fluorescence due to adherent eGFP-labelled THP-1 cells, and the right column presents a merger of HUVEC in phase contrast and the eGFP-labelled THP-1 cells (A‘-D‘). In conclusion, THP-1 adhesion on thermal damaged HUVEC is not caused through heated collagen, supporting the notion that THP-1 adhesion is initiated by the influence of heat on HUVEC. In order to determine whether the observed adhesion is restricted to THP-1 cells, we repeated this assay using primary peripheral blood mononuclear cells (PBMC) as another source of immune cells. Figure 15 E shows the quantitation of the number of adhered PBMC on non-affected and affected HUVEC after mechanical and thermal damage. Additionally, the count of adhered PBMC on untreated HUVEC and HUVEC stimulated with TNF-α is shown. The corresponding representative images are shown in Figure 15 A-F. To exclude dead-rounded HUVEC in our counting we only counted small PBMC indicated with white arrows and not larger PBMC that cannot be differentiated clearly from dying HUVEC without additional staining. Furthermore, we checked the adherence of PBMC on collagen-coated coverslips to exclude unspecific binding (Figure 15 H-J). Figure 15 G shows the highest increase in the number of adhered PBMC after stimulating the HUVEC with TNF-α but there is also a highly significant difference in the number of adhered PBMC at the adjacent area after thermal damage compared to non affected and mechanical damaged HUVEC. Also, PBMC did not show a difference in adherence on thermal damaged collagen compared to mechanical damaged collagen.To summarise, the adhesion experiments using PBMC showed more or less the same results as the experiments using THP-1 cells, indicating that the binding of mononuclear cells is restricted to thermally damaged cells in this model system.

### THP-1 adhesion on HUVEC reflects the spatial structure of differently damaged zones in the model

Given the previous observation of discrete zones of differntly damaged HUVEC (see Figure 7), we wanted to analyse to what extent the adhesion of THP-1 cells reflects the structure of differently damaged zones imposed by the geometry of the stamp.

**Figure 16:**
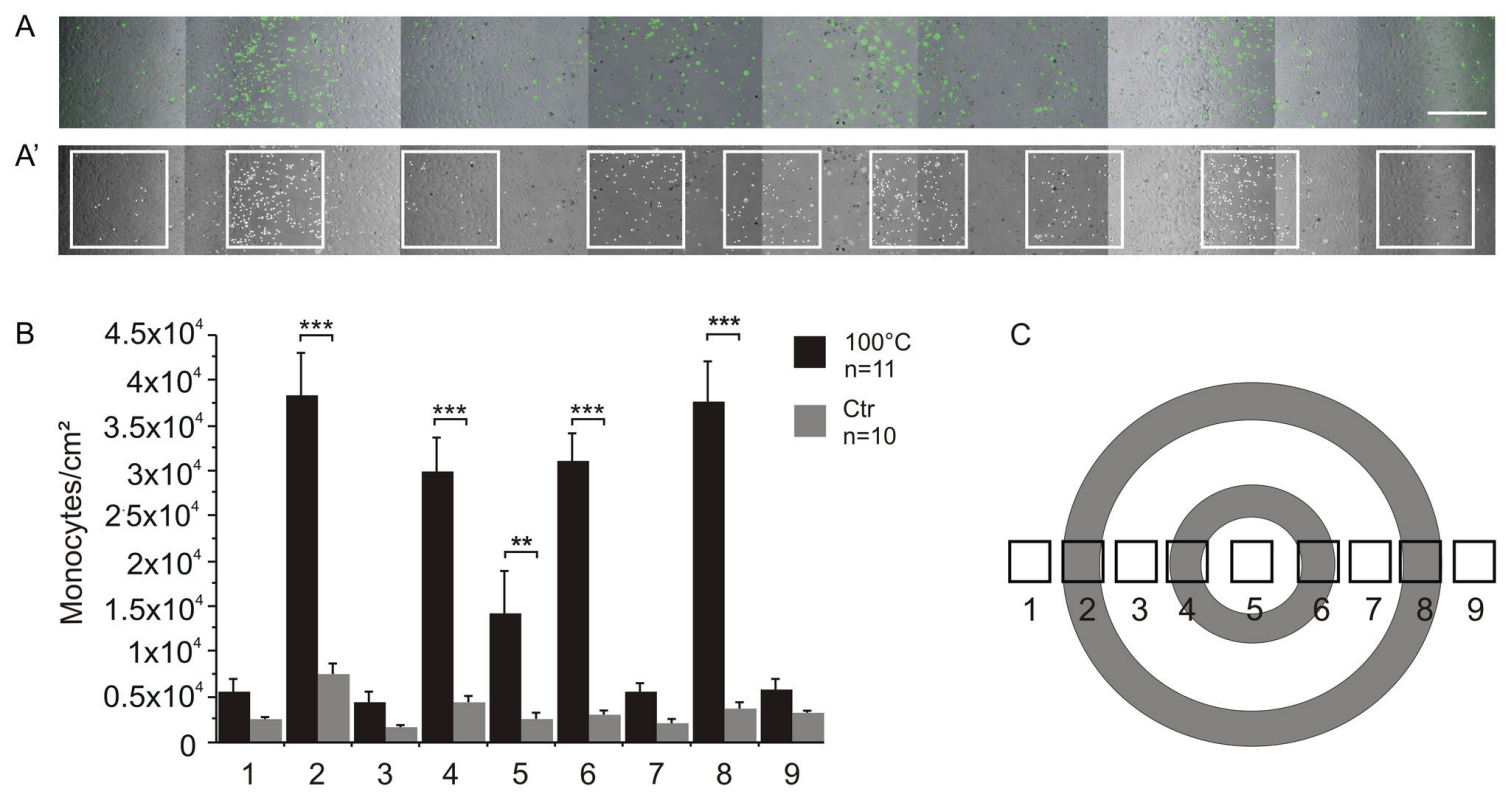
Adhesion of THP-1 on thermally and mechanically damaged HUVEC (6 h after damage). A: representative section of HUVEC (phase contrast) after thermal damage with adherent green fluorescent THP-1 (eGFP). Images were taken by fluorescence microscopy and assembled as collages to get an overview over the complete section from the left hand side to the right hand side of the 12 mm cover slip. . A’: 8-bit image of (A) for analysis 9 different areas were defined and THP-1 cells were counted. Scale bars of A and A’ 500µm. B: the number of monocytes per cm^2^ in nine different areas (showed in A’) on HUVEC after thermal damage (black bars) were determined. Areas adjacent to thermal damage (2,4,6,8) showed highly significant increase in the number of adherent THP-1 cells, whereas the unheated areas (1,3,7,9) showed no effect. Surprisingly, HUVEC in the centre (5) showed an increased THP-1 cells adherence, although most cells in this area seemed to be unaffected. The stamp control (grey bars) showed no difference in adhesion of THP-1 cells. C: schematic illustration of the stamp and the analysed areas. This experiment was performed in triplicates with a minimum of three wells per experiment and treatment. Control: n=10 images, 100 °C: n=11 images.

To this end, we scanned the cover slip from left to right and combined the detailed images in a photo-collage for further analysis. Figure 16 A shows a representative collage of thermally damaged HUVEC in phase contrast merged with a fluorescent image of the adhered eGFP-labelled THP-1 on top. The THP-1 cells were counted according to a method illustrated in Figure 16 A’: nine differently affected areas were selected, and cells were counted using an 8-bit file converted by the software ImageJ. A sketch highlighting the position of the nine different areas is illustrated in Figure 16 C. In Figure 16 B, we quantified the number of the monocytes per cm^2^ (y-scale) and plotted them against the nine different areas (x-scale, black bars). As a control, we counted the adhered THP-1 on mechanically damaged HUVEC (grey bars). The diagramme shows that there was a highly significant increase in THP-1 adhesion in the areas affected by thermal damage compared to the stamp control. Furthermore, we observed a highly significant increase in the number of adherent THP-1 in the affected areas (2,4,6,9) compared to the unaffected areas (1,3,7,8) of the same well, but only in the case of the thermally damaged HUVEC. Interestingly, we observed a significant increase of adhered THP-1 after thermal damage in the centre area (5) as well. As shown in Figure 6 C’, the cells in the centre seemed to be unaffected, as they were positive after calcein staining, indicating viability. However, we had already observed a different wound closure pattern in the centre area (see Figure 6). Together, these results strongly suggest that the HUVEC in the centre of the inner ring are more affected by thermal damage than the corresponding cells from the outer ring.

At any rate, the vital HUVEC are stimulated by the heat, resulting in an adhesion of monocytes. 

## Discussion

In this study we have established a new *in vitro* model to investigate cellular reactions in response to thermal influence, using a heat controller to allow for tight regulation of the temperature applied. As we wanted to mimic cell damage caused by electrosurgery, we selected an exposure at 100 °C for 1 sec, while at the same time applying mechanical pressure. These experimental settings most closely reflect the conditions under which electro surgical instruments are applied for laparoscopic haemostasis and blood vessel sealing[26]. We have designed and tested three different geometries for stamps, and performed further experiments with the design of two concentric rings, as this geometry allowed for an optimal ratio of damaged to unaffected cell culture surface. The presence of unaffected cells in close vicinity to cells which are thermally affected to varying degrees is a hallmark of the new model. These features set our model apart from currently used *in vitro* models that are more apt for analysis of cell migration and less so for analysing cellular damage. The most common method to study cell migration is the scratch assay [13]. In this method, cells are mechanically removed from the monolayer, leaving an area devoid of cells - the scratch. This method is well established for analysis of cell migration into the cleared area in conjunction with different treatment of the cells. A similar approach to study cell migration into an area cleared completely of cells has previously been described by 27. Here, the cells are cultured on an elastic film containing holes, which is positioned onto the cell culture plastic surface. This film serves as a kind of stencil, and upon removal of the film, only the cells in the holes remain, and they spread out into the cell-free surroundings. The complete removal of cells or cell debris is desirable inasmuch as the cell migration is not to be affected by remaining debris. In contrast, in our model cellular debris remains, which more closely reflects the situation found in a burn wound. The significant improvement of our model system over the scratch assay lies in the fact that thermal damage can be integrated, resulting in a more complex response of the affected cells, which otherwise could only be mimicked by additional application of stimulants such as TNF-α.

A somewhat different approach is taken in the case of the Electric Cell-substrate Impedance Sensing (ECIS®) [28,29]. A precise “wound” on the cell monolayer is inflicted by the application of a high-frequency alternate current. The wound is restricted to a 250-µm-diameter electrode, and cell debris is not actively removed. Concerning the small wound size, the wound closure is faster compared to wounds generated by a scratch, and cell division can be excluded during migration studies. The improvement on the scratch assay lies in the easy and precise quantitation of wound closure by online measurement of the impedance of the cell monolayer, which is indicative of the degree of confluence. Despite this, cells become quantitatively detached within the damage zone (our own observations). As the temperatures reached by this method are not well analysed, this leaves open to uncertainty the question to what extent cell death is a consequence of the electric field and to what extent it is a consequence of thermal damage. Given that the temperature cannot be controlled, this system is inadequate for the analysis of thermal damage. Rather, as is the case with the scratch assay, this method is widely applied to investigate cell migration, again in the context of different treatment of the cells, mostly for chemical compound screening. Concerning thermal damage, a variety of protocols have been developed to expose cells to well-controlled temperatures above body temperature, notably in the range from 42 °C and 50 °C [30,31]. In these protocols, entire cell cultures are exposed to a transient, non-lethal elevation in temperature for a few minutes up to an hour in order to investigate the heat shock response, a naturally occurring protective stress response to counteract mild body overheating. As body-overheating is systemic, a spatial differentiation in affected and non-affected cells is not required in these settings. Sobral et al.[32] have established an experimental burn model on human keratinocytes cultured on matrices containing, among others, collagen and Matrigel™. They used stainless steel rods from 1.25 to 5 mm in diameter heated to 170 °C to produce the burn defect; the high temperature was chosen to compensate for any loss of heat to the environment. However, the actual temperature applied to the cells was not carefully determined. Whereas the consequence of contact time or contact area on burn defects was analysed, the cellular responses elicited were not studied to any detail. In contrast, the model system discussed here allows for a precise regulation of the temperature applied to the cells because of the integrated temperature control. Furthermore, loss of temperature by conduction is minimised by insulation.

The spacing of the two concentric rings (2.3 mm) was selected in such a way as to achieve an optimal distribution between affected and unaffected areas within the boundaries of a 12 mm cover slip. Too narrow a distance between the contour elements (e.g. a 1-mm spacing) resulted in irrevocable damage of the entire cell layer, whereas too wide a distance would have unnecessarily limited the extent of the area damaged. For the application in larger cell culture vessels, such as the six-well format, the geometry could be modified by the introduction of a third concentric ring, based on the optimal spacing determined above.

As during electrocautery, the majority of affected tissues are blood vessels, we investigated the fate of endothelial cells. Viability staining revealed that HUVEC in the adjacent area of thermal damage were highly affected, whereas the surrounding cells were unaffected. We performed a migration assay to prove that the surrounding cells had retained their ability to migrate into the wounded area. This demonstrates that our model is suitable for further analysis of the surrounding HUVEC after thermal or mechanical damage. Due to the differently affected zones after thermal damage, extending from vital cells to dead cells, we supposed that some cells are undergoing necrosis, a form of cell death in which cells first swell, and after the plasma membrane collapse, the cells lyse [33] whereas others might undergo apoptosis. The typical morphology of cells undergoing apoptosis has been previously described by 34 as “characteristically scattered single cells that are manifested histologically by the formation of small cytoplasmic fragments” and add that the nuclei of apoptotic cells appear “condensed or fragmented”. Additional descriptions of the morphology of apoptotic cells are given elsewhere [17,18]. We detected both the cytoplasmic fragments (as shown in Figure 7 C, C’’and C’’’) and the condensed form as well as the fragmented form of nuclei (data not shown) in the zones affected after thermal damage. Apart from the visual interpretation of the morphology of the affected cells, we could prove the presence of both types of cell death through additional stainings. Compared to necrotic cells that were present in the entire damage zone, only a few cells at the area adjacent to thermal damage showed caspase-3 activity, indicating that these cells underwent apoptosis. Additionally the number of cells stained positively for cleaved caspase-3 declined dramatically from three hours after damage to six hours after damage. We suppose that these dying cells are stimulating the surrounding vital cells. Compared to the thermally damaged cells, only few cells were affected after using the stamp unheated.

As previously described [35], reactive oxygen species (ROS) are generated shortly after cells are stressed, in a process controlled by a constitutively active form of Rac GTPase. The cellular ROS level increases continuously and reaches its maximum approximately after one hour, followed by a quick decrease. Therefore, we determined ROS generation to judge whether cells are stressed due to the heat. In the cells immediately adjacent to the dead cells, we observed an increase in ROS levels after 30 min, which continued up to one hour. Two hours after thermal damage, the ROS levels varied substantially. Apparently, ROS formation is an integral part of the early response of affected but viable cells. To what extent these cells survive later on could not be followed -up due to technical constraints.

Further common markers to study cell stress are heat shock proteins. The stress response maintains the activation of heat shock gene expression whereby cellular mechanisms to protect organisms against various physical and chemical stresses, including elevated temperature, heavy metals, toxins and oxidants are activated [36]. On the one hand, Hsp 27 for example functions as antioxidant, lowering the levels of ROS. On the other hand, it is involved in the protection from programmed cell death by inhibition of caspase- dependent and mitochondrial apoptosis, but additionally from other types of cell death as well [22]. Because we could already show that apoptotic as well as necrotic cells are limited to the adjacent area of thermal damage, we were interested whether there are additional factors that might protect the cells from cell death. Therefore, we analysed the expression of two heat shock proteins with a different kinetic: Hsp 27 is activated by phosphorylation and relocalises from the cytoplasm to within or around the nucleus [37]. Heat shock activates Hsp 27 within minutes [37], and according to Haddad et al. 2001, phosphorylated Hsp 27 reaches a peak after 60 min after treatment of fetal alveolar epithelial cells with lippopolysaccharide (LPS), followed by a decrease [23]. The treatment of prostate cancer cells (PC3) with transforming growth factor β (TGF β) induced phosphorylation of Hsp 27 more or less in the same time-dependent manner [38],reaching a peak 90 min after treatment. After thermal damage using the two-ring stamp, we could also detect a relocalisation from the cytoplasm to the nucleus within1 h after thermal damage but not after 24 h, in line with the activation studies of Hsp 27 discussed above. In contrast, Hsp 70 was not expressed after 1 h but after 24 h. The Hsp 70 expression kinetic is different from that of Hsp 27, as has already been described by Wang et al. 2003, who performed a kinetics study of the endogenous Hsp 70 expression after different durations of heat shock in bovine aortic endothelial cells [24]. Taken together, we could detect the activated heat shock proteins pHsp 27 and Hsp 70 locally at the adjacent area of thermal damage, with a kinetic in agreement with the data published by others. These results demonstrate that our *in vitro* model correctly reflects the heat-shock induced survival mechanisms.

Because we could detect ROS synthesis in the same areas where we detected heat shock proteins, we suppose that their expression is ROS-dependent, and should be further analysed. The presence of heat shock proteins leads to the assumption, that some cells at the adjacent area might be protected from cell death. This hypothesis is strengthened by the observation that in the apoptosis and necrosis staining additional unstained but morphologically affected cells are present in these areas (see Figure 8, arrow 4). Our model is therefore suitable for further investigations of thermally affected cells, as no additional stimulations are needed.

A hallmark for an ongoing process of wound healing is the adherence of leukocytes, neutrophiles and monocytes to the endothelium. Activated endothelial cells express selectins and Ig-like surface proteins to enable the adherence of inflammatory cells, which is a prerequisite for the migration of inflammatory cells to the site of inflammation [10,12]. To analyse whether mononuclear cells are able to recognise thermally damaged HUVEC, we performed adhesion experiments using HUVEC and leukaemic monocytes (eGFP-labelled THP-1) as well as HUVEC and primary peripheral blood mononuclear cells (PBMC). We could see a remarkable difference between the numbers of adherent mononuclear cells to thermally damaged HUVEC compared to mechanically damaged HUVEC. Furthermore, the mononuclear cells only adhered locally to cells adjacent to the thermal damage zone but not to the surrounding unaffected HUVEC, implying that mononuclear cells specifically recognise thermally affected cells. As our data show, mononuclear cells bound not only in the centre of thermal damage where only necrotic cells are found, but they also adhered at the areas adjacent to thermal damage. Only a few apoptotic cells were present in these adjacent areas, and their number decreased until 6 h after thermal damage, whereas the adhesion of THP-1 cells increased until 6 h. As monocyte adhesion is at least partially mediated via integrins, we tried to detect the corresponding ligands VCAM-1, ICAM-1, and E-Selectin on the surface of HUVEC. In preliminary immunofluorescence studies we could detect a faint signal of VCAM-1 expression in a few cells 6 h after thermal damage, but not after mechanical damage, whereas we could not see an increase in ICAM-1 or E-Selectin expression (data not shown). As previously described, VCAM-1 expression is related to ROS-dependent signalling [39,40]. Whereas Marui et al. described that ICAM-1 and E-Selectin expression is regulated ROS-independently; contradictory mechanisms have been reported elsewhere [40,41]. An ICAM-1-related-PMN-adhesion on HUVEC was observed after ROS stimulation without a visible increase in ICAM-1 expression [41]. Possibly, the amount of proteins at the cell surface was below our limit of detection. At any rate, the number of VCAM-1-positive cells is too low to explain the mononuclear cell adherence, so we must assume that these cells also adhere to dying or dead HUVEC, although not to heat-treated collagen.These data support the hypothesis that ROS may play a major role in cellular response to thermal stress, but a more detailed study would have to be made to elucidate this. 

In conclusion, our model is suitable for investigations of cellular responses to thermal influence *in vitro*. The damage generated by our model is highly reproducible and locally confined. This set-up could easily be expanded to induce locally confined frostbite damages by integrating a Peltier element. We have demonstrated that thermally damaged cells in monolayer cultures showed different cellular response compared to merely mechanically damaged cells. Although we restricted our experiments to HUVEC, we would assume that our model is also suitable for other cultures, e.g. 3D cultures. This would extend the applicability to skin models as well, bridging the gap to *in vivo* cutaneous wound healing models [42] or high voltage electrical burn models [43].
